# Modelling household well-being and poverty trajectories: An application to coastal Bangladesh

**DOI:** 10.1371/journal.pone.0238621

**Published:** 2020-09-04

**Authors:** Attila N. Lázár, Helen Adams, W. Neil Adger, Robert J. Nicholls

**Affiliations:** 1 Department of Geography and Environmental Science, University of Southampton, Southampton, United Kingdom; 2 Department of Geography, King’s College London, Strand Campus, London, United Kingdom; 3 Geography, College of Life and Environmental Sciences, University of Exeter, Exeter, United Kingdom; 4 Tyndall Centre for Climate Change Research, University of East Anglia, Norwich, United Kingdom; Sveriges landbruksuniversitet - Campus Umea, SWEDEN

## Abstract

Resource-based livelihoods are uncertain and potentially unstable due to variability over time, including seasonal variation: this instability threatens marginalised populations who may fall into poverty. However, empirical understanding of trajectories of household well-being and poverty is limited. Here, we present a new household-level model of poverty dynamics based on agents and coping strategies–the Household Economy And Poverty trajectory (HEAP) model. HEAP is based on established economic and social insights into poverty dynamics, with a demonstration of the model calibrated with a qualitative and quantitative household survey in coastal Bangladesh. Economic activity in Bangladesh is highly dependent on natural resources; poverty is widespread; and there is high variability in ecosystem services at multiple temporal scales. The results show that long-term decreases in poverty are predicated more on the stability of, and returns from, livelihoods rather than their diversification. Access to natural resources and ecosystem service benefits are positively correlated with stable income and multidimensional well-being. Households that remain in poverty are those who experience high seasonality of income and are involved in small scale enterprises. Hence, seasonal variability in income places significant limits on natural resources providing routes out of poverty. Further, projected economic trends to 2030 lead to an increase in well-being and a reduction in poverty for most simulated household types.

## Introduction

The eradication of poverty is a core target of the Sustainable Development Goals [SGDs, 1]. However, the SDGs further highlight that poverty reduction should occur without undermining the natural environment and resource base. Trade-offs between poverty reduction and natural resource management scale down and play out at local level. Thus, meeting the SDGs requires understanding of household dynamics and the contribution of natural resources to household incomes, analysed in the context of wider biophysical processes that affect ecosystem service functioning and provision, especially under climate, demographic and economic change [2]. Natural resources are more than just income sources: they represent safety nets and thus essential elements of well-being for dependent populations [3, 4].

It is well established that poverty, defined here as the absence of material well-being but with multiple social and health dimensions, is not a permanent or inevitable state for individuals and households, but rather a transient and dynamic state, with high temporal variability [5]. Yet poverty can be persistent over whole lifetimes, and whole populations can be trapped in poverty, or fall into and escape conditions of poverty many times over their life course [6]. Cross-sectional assessments of poverty are limited in identifying the transitionary nature of poverty, especially for populations and places with seasonal and multi-annual cycles [5, 7]. Temporal changes in poverty status are usually identified through longitudinal studies carried out years apart [e.g. 5, 8]. Yet these transitory patterns may also occur over shorter timescales driven by seasonal dynamics [9, 10]. Idiosyncratic shocks such as death of a family member or covariate shocks that affect the whole community (e.g. floods and cyclones) invariably lead to a deepening of poverty [5, 7, 11, 12]. It is also established that descent into poverty can also occur gradually through the culmination of multiple pressures, such as increased food prices, school fees, and healthcare expenses [7]. Erosive coping strategies can also create an adverse cycle of decline towards poverty.

Seasonal patterns of work and variable ecosystem productivity for resource-based economies have been shown to cause households to move into, and out of, poverty [13]. Seasonal lean periods before agricultural harvests, or when resources such as fish are not available, reduce consumption and expenditure [14]. This is compounded when households are unable to save or store in order to transfer assets between the seasons, and affects households without access to natural resources through an increase in food prices [15]. Thus, it has been widely shown that households diversify their livelihoods through the year to smooth income across seasonal low periods and spread risk [16], and strategies include off-farm income sources, and remittances from family members [17–20]. However, livelihood diversification can be limited by a lack of capital [21] and by the availability of livelihood opportunities that do not undermine well-being or place households in equally precarious situations [7].

Models provide an approach to better describe and understand how poverty evolves and project its dynamics under future conditions, including the impact of different policy choices. The mechanisms by which poverty becomes entrenched are characterised in economic models as convergence on an equilibrium that is below a poverty threshold, caused by a lack of technology and/or institutional, or geographical and environmental constraints [6]. At the household level, these technological, institutional and environmental constraints are manifest as lack of access to land or other natural resources, social exclusion, and lack of credit [22, 23]. Economic models also estimate the effects of policy interventions or as external constraints on levels of poverty for households [24, 25]. However, empirical models have limited ability to provide an insight into mediating factors because they use associations rather than casual links. Furthermore, empirical models are often highly aggregate providing population perspectives at coarse spatial scales. Finally, empirical forecasts are data driven as opposed to process-driven, questioning their validity if circumstances change considerably. At the other end of the spectrum, agent-based models (ABMs) consider small-scale processes, focusing on individuals, households or villages, including the agent-agent interactions and hence provide a bottom-up approach. Only the MP-MAS model was found in the literature that focused on simulating poverty-environment linkage (Schreinemachers, Berger et al. 2007, Schreinemachers and Berger 2011). They used an ABM to estimate poverty for agriculture systems linking environmental change with economy-based farm management decisions and estimated poverty through calorific intake. Their landscape model operates at 10-100m resolution requiring spatial detail on local selling-buying prices, individual household composition, and available resources. The calorific intake calculation is based on a user defined income parameter, instead of being calculated from household expenditure, thus despite the model comprehensiveness and complexity, the poverty estimation is very simple.

Hence, we developed a new intermediate hybrid modelling approach–the Household Economy And Poverty trajectory (HEAP) model. HEAP draws on and combines elements of ABMs and empirical approaches and in this paper is validated and demonstrated in coastal Bangladesh. HEAP couples biophysical and social sciences, provide process understanding at sub-annual temporal resolution, and analyses poverty under dynamic conditions that can inform policy. The model is designed to be integrated with environmental and economic changes to dynamically simulate household poverty and health outcomes through the consideration of a wide range of coping mechanisms. The principles of HEAP are established from the poverty literature, supplemented and tested by qualitative surveys in the study area. This is combined with a large quantitative survey in the study area to define the household agents and their characteristics. The model focuses on coping strategies such as savings, loans, expenditure levels and kinship support, to maintain and improve the quality of life in response to external conditions.

The aim of this paper is to investigate (i) the association between seasonal variation in livelihoods and incomes and the poverty trajectories and (ii) the role of natural resource-based livelihoods in reducing the incidence of poverty over time. In line with well-established approaches, we measure poverty as a threshold below which a household does not have the capability to have adequate expenditure for food, education, healthcare, etc. [26].

The paper first introduces the modelling approach and the study area, discusses how household-level strategies are conceptualised within the HEAP model and the principles that have guided its design. Then a technical overview of HEAP and validation results are presented. To illustrate different results, we present an analysis of seasonality of income and poverty of different household archetypes expressed through expenditure, and show the impact of seasonality on multi-dimensional poverty trajectories from 1990 to 2030. Finally, we discuss the results and conclude. The Supporting Information document gives the full details of model design and governing equations.

### Geographical context of coastal Bangladesh

**Fig 1:**
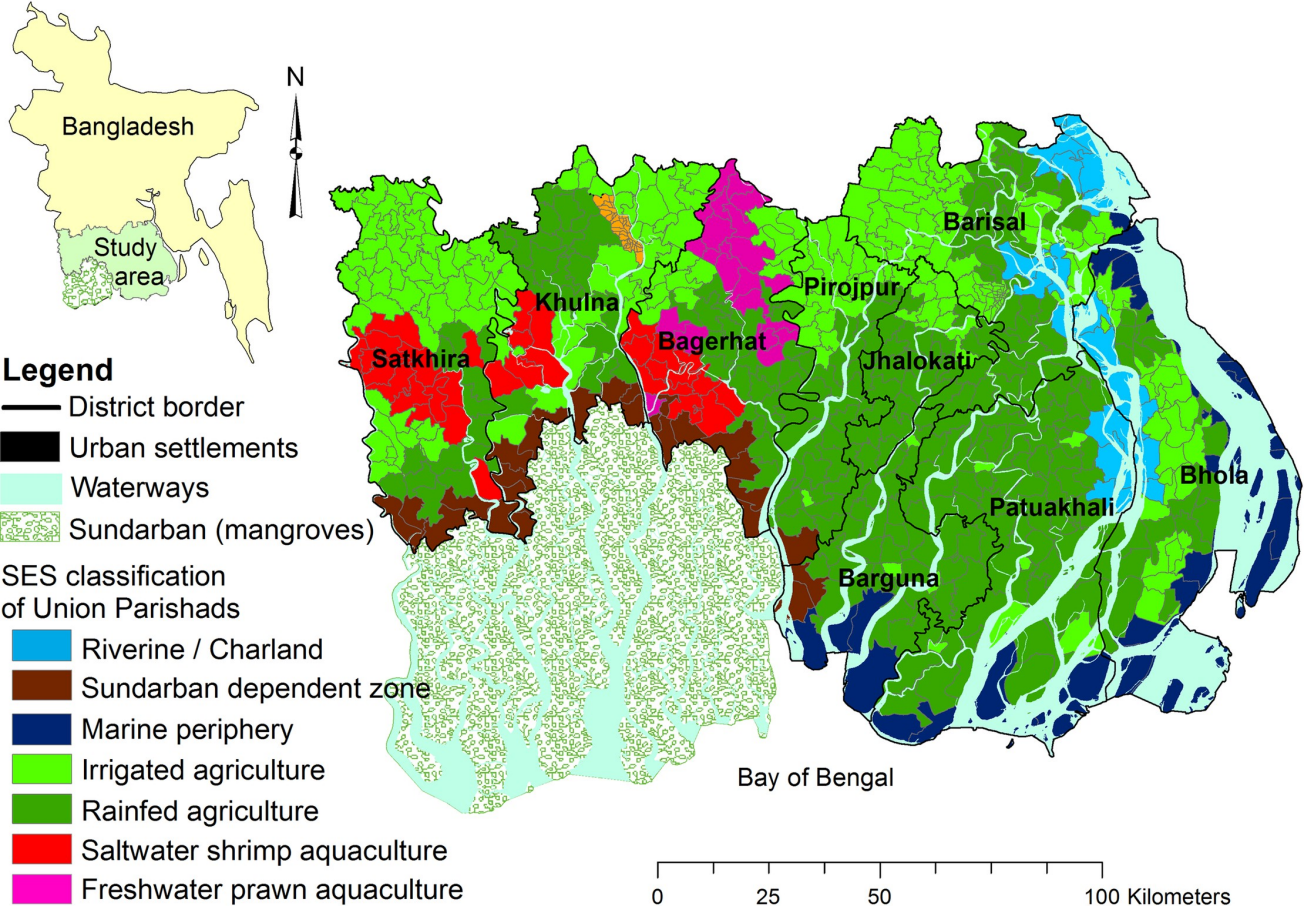
Study area boundary in Bangladesh and the Social-Ecological System (SES) classification of the Union Parishads for 2014.

The model is calibrated using the example of resource-dependent households in the southwest and south central coastal zone of Bangladesh (Fig 1). The population is 14 million, with 653 Union Parishads (unions from hereon) and one large city, Khulna. Unions are the local council units in Bangladesh with, on average, 26 km^2^ surface area, nine villages and 21,000 people. The dominant land use is agriculture, rice being the principal crop. Shrimp cultivation–both saltwater variety (Bagda) and freshwater variety (Golda), has become widespread in the region over the past two decades. The collection of forest goods from the Sundarbans, the world’s largest mangrove forest, also provides an important livelihood for the poor who have access [27].

Environmental and natural resource trends (1950–2010) in the study area are summarized by Hossain, Dearing [28]. Dry season river and soil salinities have shown a two to ten-fold increase since the 1970s, impacting dry season agriculture. Wet season salinity levels have decreased due to increased precipitation and high river flows. Despite this, agriculture productivity (e.g. rice, vegetables and spices) have steadily increased over this period. However, industrial crops (sugarcane, jute) and potato have declined, and since 2007/8, the major rice varieties also experienced significant reductions in yield (except the high yield variety of wet season rice). Pond-based aquaculture productivity also increased, but natural fisheries are generally declining. Generally, embankments and polders provide coastal protection in the study area. The Sundarbans mangrove forest also provides wave and surge attenuation, as well as coastal erosion reduction, fish nurseries, biodiversity maintenance, provisioning services (e.g. timber), air purification, climate regulation and cultural services [29].

The national incidence of poverty has reduced by about 25% since the 1990s and the current incidence of poverty in Khulna Division and Barisal Division are 31 and 39 percent, respectively [30]. Land distribution dating back to colonial periods [31] resulted in highly uneven landownership in the region with a few large landowners (3% of households) in proportion to the high numbers of small landowners and functionally landless (54% of households, owning only 17% of land) [32]. Land consolidation is further intensified through land grabbing and expropriation [33, 34]. Patronage and elite control is endemic in Bangladeshi villages, as the poorest households trade their voice, independence and right to be involved in decision-making on village matters, for the safety nets provided by village elites [35]. Micro-credit is ubiquitous across Bangladesh but does not prevent informal money lending [36]. Many households have some form of loan and for those without capital to act as collateral, high interest rate informal loans further increase their vulnerability.

## Methods

HEAP is integrated, dynamic and process-based. HEAP aims to simulate the consequence of income fluctuations on the quality of life (i.e. different expenditures) and thus on the seasonal and multi-decadal poverty trajectories of rural households.

### Model structure and assumptions

The model structure is based on fundamental principles of poverty studies used to create a quantitative and dynamic model, with predictive capacity, linked to environmental, demographic and economic drivers of change. There are four key assumptions. First, poverty is multi-dimensional, and in this application we operationalise this concept by incorporating expenditure, health and education [14, 37]. Second, decisions on risk spreading and income diversification are made at the household level and thus the household is the key unit of analysis [38, 39]. Third, levels of poverty among households in the same localities and communities are unequal and that inequality constraints the coping strategies of poorer households [5, 7]. Fourth, poverty can be transitory, showing inter- and intra-annual variability [13].

The model incorporates three additional assumptions concerning how households respond to adversity. These are common in many low income, marginalised, natural resource-dependent societies and are also apparent from initial in-depth household interviews in the study area [17, 40, 41]. First, households spread risk by diversifying livelihoods across multiple income sources, often changing by season, and using migration to access them [38, 42]. Second, the model considers social and financial constraints on coping strategies–not all coping strategies such as access to savings or credit are available to all households, and households may use erosive strategies in order to maintain their social standing and meet social norms [43]. As examples, households take loans to pay for dowries and weddings; landowners refuse to take on wage labour because of the impact on their community status; and traditional fishermen may refuse labouring work on cultural reasons. Third, where households have a range of coping strategies available to them, they will show a preference for those that do not undermine future well-being [44]. The relatively wealthy with assets and extensive social networks through membership in local institutions (e.g. village council) can easily enter high-end off farm activities [7, 45], whereas the poorest, landless farmers, tend to have access to only low-end and exploitative off-farm opportunities (e.g. construction, maid, rickshaw pulling) that undermine future resilience [13].

During shocks, better-off households have more to lose in absolute terms, but they have a greater range of adaptation options [7] being able to draw on cash savings, credit and food stores [11]. However, not all households have access to credit due to the lack of collateral, social networks or steady income [40, 41] and, as such, they turn to informal money lenders [7]. The lowest wealth quintile consistently misses out on development efforts, when the second and third quintiles have improved on multiple poverty indicators [45].

### HEAP model implementation

The key features of the HEAP model are as follows:

- Agent-based-type model structure
- Sub-annual resolution to capture poverty transitions
- Realistic household characterisation, with seasonal livelihood behaviours
- Expenditure-based outputs to capture changes in quality of life as an indication for poverty
- Explicit simulation of coping mechanisms, including loans and kinship support to smooth income variation and mitigate shocks
- Optimisation of household behaviour to maintain and improve quality of life.

These are discussed in more detail below.

The spatial resolution of HEAP is at the level of the union (N = 653), and has a monthly temporal resolution. HEAP considers two hierarchical levels: unions and archetypal households. Unions are characterised by their land use composition and location (Fig 1) following a socio-ecological system classification [41, 46]. This classification is assumed to define the livelihood opportunities and thus the presence of the 36 archetypal households in a union. Each household archetype is assumed to be representative of an average household of its type within the specific union.

**Fig 2:**
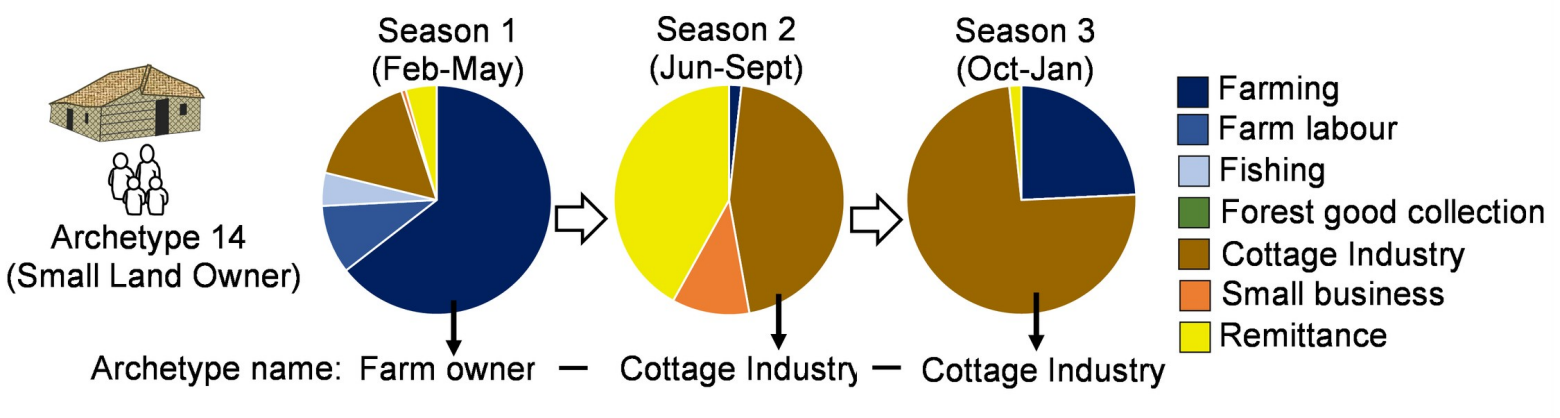
An example of the archetypal household classification. The pie charts shows a household’s seasonal income distribution. The archetype grouping and the name of the archetype is based on the income that dominates in each season. For example, this small land owner archetype 14 is described as Farm Owner–Cottage Industry–Cottage Industry.

In HEAP, the archetypal households represent different household diversification strategies. Based on the seasonal household survey of Adams, Adger [46], we identified 20 dominant archetypal households, representing 68 percent of the 1478 surveyed households. Seven main livelihood categories were observed: (1) Farm owner (agriculture and/or aquaculture including sharecropping); (2) Farm labourer; (3) Fisher; (4) Forest goods collector (timber and non-timber products); (5) Cottage industries (i.e. small scale production); (6) Small service- oriented businesses and salaried jobs; and (7) No job (i.e. student, unemployed and retired). Based on the longitudinal observations, each household was then characterised by their seasonal variation in dominant income source and access to land (Fig 2). Intra-community inequality is considered by differentiating the household archetypes by levels of land ownership (landless or homestead: <0.5 acres, small land owner: 0.5–2.5 acres, large land owner: 2.5+ acres), expanding these 20 basic types into the final 36 household archetype categories (not all archetypes are represented in all three land size categories (Table S1.1)).

**Fig 3:**
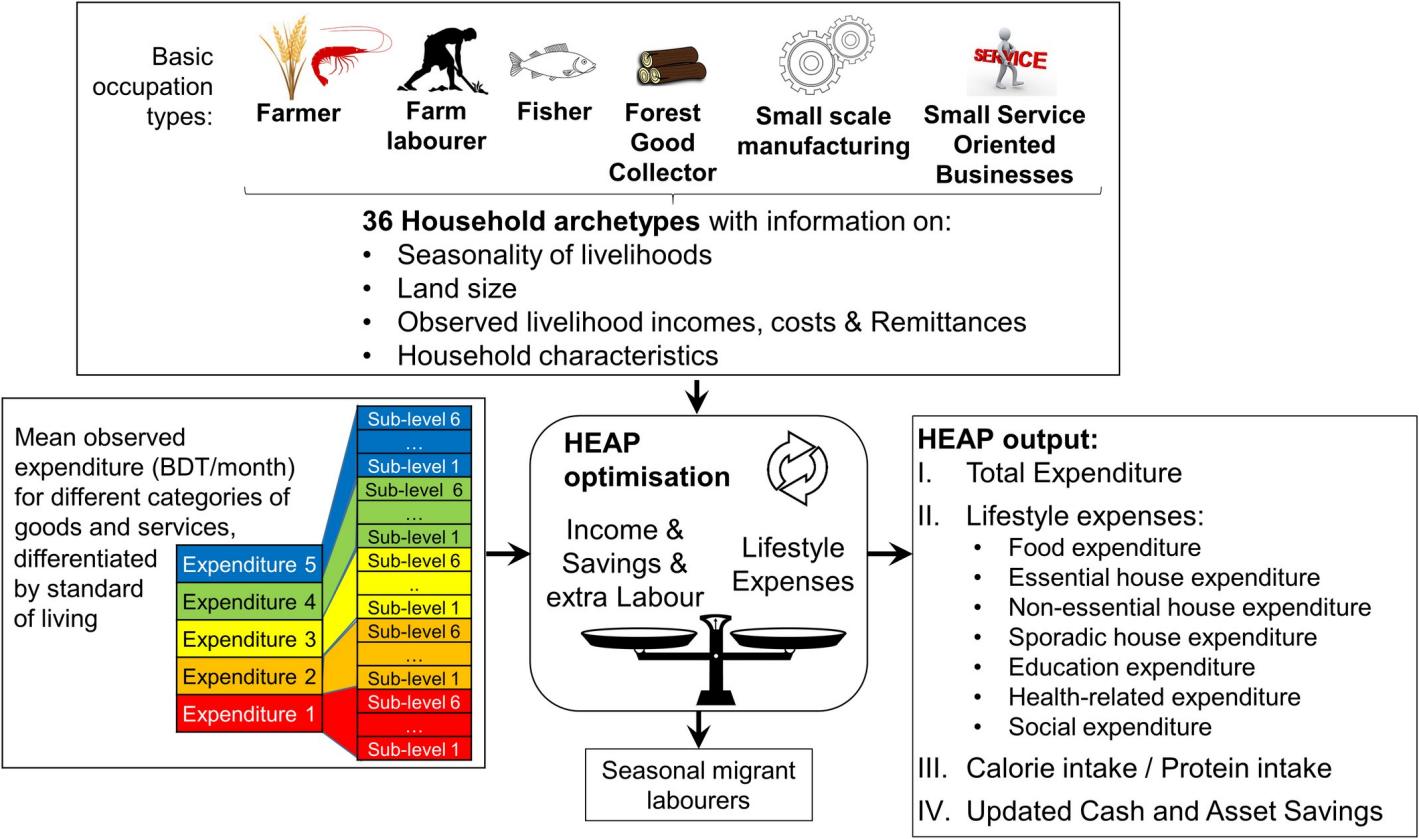
The HEAP model components.

HEAP simulates the economic mass-balance of archetypal households by month and union (Fig 3) considering all income sources, the typical levels of expenditure, household characteristics such as land size, and levels of subsistence:

$$\begin{array}{l} Residual\;Income=Total\;Income+Total\;Savings–Fixed\;Expenditures=\\ \;=Total\;Livelihood\;Income+Remittances+Loans+Cash+Assets–\\ \;Livelihood\;Expenditure–Loan\;repayments \end{array}$$ (1)

*Total Income* includes *Livelihood Income* (farming, fishing, off-farm, etc.), *Remittances*, and if a loan was taken, the amount of the *Loan*. *Total Savings* include *Cash* and *Asset* savings. *Fixed Expenditures* refer to expenses such as *Livelihood Expenditure* (e.g. seeds, labour hire) and *Loan repayments*. The *Residual Income* can be used to pay other expenses around the household, which we categorise into five groups depending on the need, cost and frequency of expenditure: (i) food, (ii) day-to-day expenses (e.g. cooking fuel, electricity), (iii) non-essential expenditures (e.g. clothing, furniture), (iv) sporadic expenses (e.g. house repairs, assets such as tractor), (v) education, (vi) health and (vii) social expenditures (e.g. marriages and funerals). Out-of-pocket payments for healthcare can push households onto a negative poverty trajectory [47]. Such shocks are unpredictable and sporadic and thus HEAP considers this as a flat monthly fee, scaled to overall expenditure levels. *Remittances* from migrant household members are also included, because migration is an important risk spreading strategy that also allows investment in livelihoods [38, 48].

The simulated types of expenditures represent different basic needs, costs and frequency. The model is based around income over expenditure, because expenditures (i) indicate whether minimum basic needs are being met, (ii) allow the model to consider the effect of loans and savings on fluctuations in income, (iii) show whether households are able to survive periods with no income and (iv) allow the calculation of important poverty indicators [49]. Rural inequality is built into the model by including expenditure quintiles that to represent different levels of quality of life. HEAP uses an income and expenditure calculation to simulate the financial resources available to meet a certain standard of living (i.e. expenditure levels). Within each expenditure level, six strata of expenditure are created (i.e. sub-levels) to allow for a gradual reduction in the quality of life as expenditure levels fall due to sustained reduction in income. However, households use coping strategies to maintain their quality of life if income drops.

If households can meet their well-being target (i.e. expenditure level), no further coping strategies are used. If their target is not met, and coping is not sufficient to maintain their current expenditure, the household falls to a lower expenditure level (see Fig S1.5). Conversely, prosperous households can increase their quality of life and move up an expenditure level when they meet the criteria (sufficient finances for a specified waiting time). The model optimises the expenditures and coping strategies to maintain and, if possible, improve the level of expenditure of the household. In order to understand how expenditure translates into well-being, we separately estimate expenditure on food, health and education, following the approach of Alkire et al [50]. Further, by having a set target expenditure levels and coping strategies amongst the model parameters, we cover the aspirational elements of well-being.

HEAP aims to minimise the number of members engaging in labour work (*m*), minimise the number of coping strategies (*cs*), but maximise the financial capacity (total income–total expenditure; *inc—exp*) while maintaining a minimum net savings (e.g. 10 percent; see section S4.4 for more details):

$$\max\sum_{m\in M}\;\sum_{cs\in CS}\max\{0.9*\mathrm{inc}(t)-f(\exp,t),0\}$$ (2)

The model parameters are informed by data from a bespoke seasonal household survey in coastal Bangladesh [46, 51], qualitative fieldwork in the study area [46] and secondary data from Bangladesh [e.g. 7, 13, 30, 45, 52, 53]. The initial household values were not calibrated because this is not practical for 653 unions and 36 household types in each union. Rather they are set as the mean observed value for all unions and archetypes. Initial setting of household asset and cash savings are less important, because coping actions based on the imbalance of aspirations, incomes, expenses and savings, and the model normally settles similarly within 5–10 years (i.e. an equilibrium is reached). Thus, we started the simulation in 1985 and the first six years are excluded from the results [54].

In this application, all inputs are based on observations drawn from the HIES datasets [30, 55, 56] and a dedicated seasonal household survey [46]. As sufficient spatial detail could not be extracted from these datasets, the livelihood incomes of the archetypes are based on the observed mean regardless of the geographic location, rather than being differentiated by the quality and availability of different biophysical resources. To estimate a continuous monthly input dataset, the observed seasonality within the household survey (taken in 2014) was superimposed on the annual HIES datasets and the gaps between the time slices (1991, 1995, 2010, 2014) were filled by interpolation. To extend the simulations to 2030, we assumed Business As Usual (BAU) economic trends for all sectors and livelihood expenditures (Table S1.3). The BAU scenario was created using stakeholders elicitation methods [57], which were translated into economic values [39]. Using these percentage changes, the observed seasonal incomes and expenditure levels were linearly increased or decreased between 2015 and 2030. The BAU scenario narrative assumes that population levels do not change significantly (although projections indicate decline beyond 2040 [58]), intensive land use practices result in increased land degradation and yet, income levels increase due to rapid developments in communications and market access. Thus, land degradation, technological change and general equilibrium evolution of relative prices are already considered in the economic assumptions and these only play a role in the HEAP simulations through the income timeseries.

HEAP has a number of limitations and assumptions. Currently no stochasticity is considered (e.g. cyclone landfall). Land holdings are equiproportional based on aggregate demographics and land cover changes in a union. Productive asset accumulation is part of the simulated total asset value. The model currently does not allow the household archetypes to change livelihoods, because adaptation decisions are complex and beyond assumptions on farm-management. There are two interactions between the different archetypes: (i) competition for farm labouring jobs within a union, and (ii) the defined minimum farm size might force some farm-owner households to off-farm livelihoods. Households do not make predictions about their long-term prospects or the likely future effects of their chosen coping strategies (e.g. sell assets), they simply react to the present circumstances. Thus, HEAP shows the well-being potential of different livelihood approaches. The only hard coded assumption of HEAP is the uptake of labouring jobs to augment income. Thus, even though HEAP was developed and set up for coastal Bangladesh, it can be applied in other rural developing areas by setting appropriate coping preferences and model parameters (Table S1.2).

### Model validation

To validate the model, monthly and household-level simulation results were aggregated to union level and then to the study area level by calculating the minimum, mean, maximum and standard deviation. Finally, these statistics were plotted with the observations to qualitatively validate the model behaviour (i.e. mean values are close to the observations, they follow the same trend and the observations are within the simulated range). Quantitative validation of such a detailed model as HEAP is difficult, because monthly observations are not available at the scale required.

**Fig 4:**
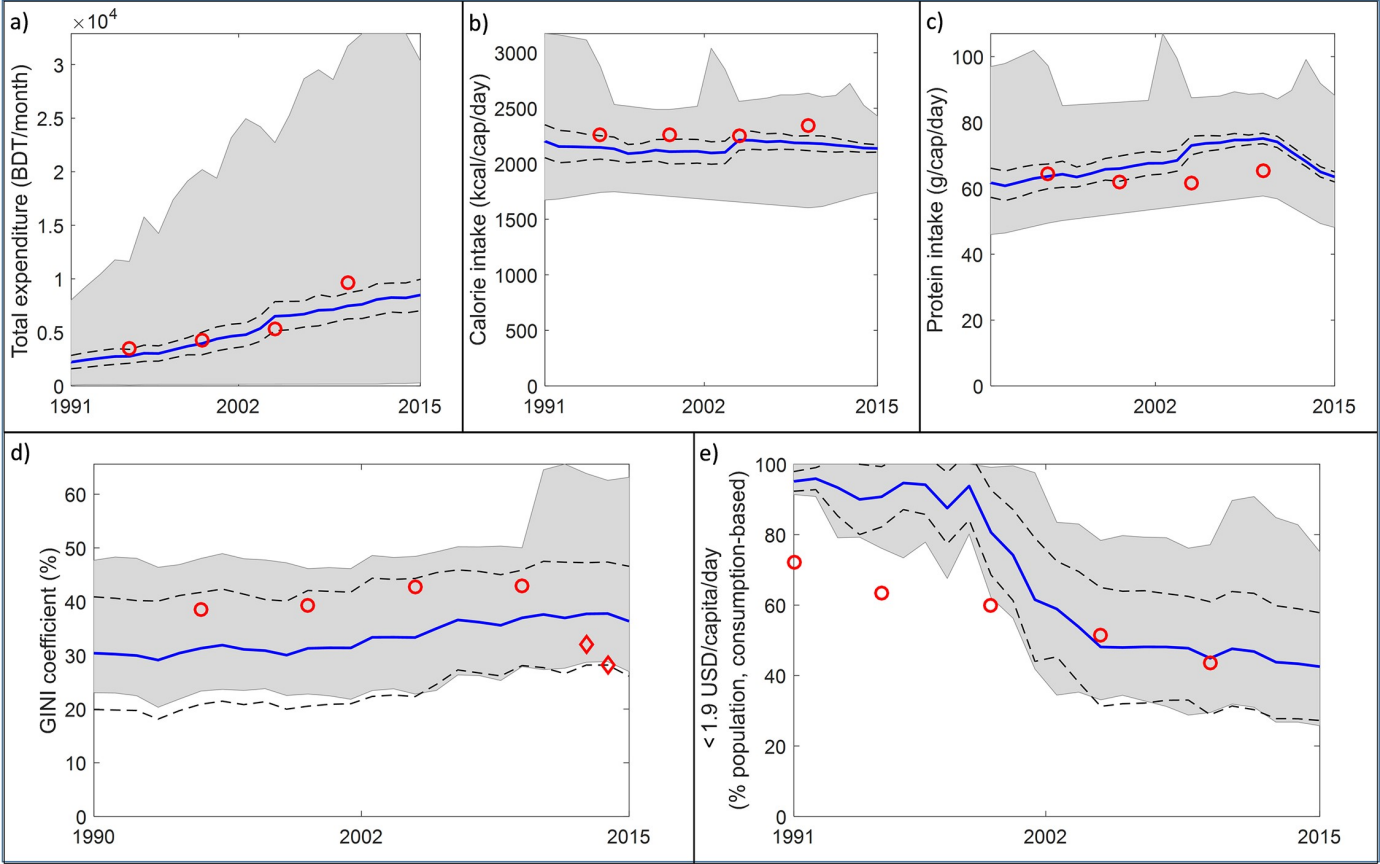
Validation plots showing (a) total expenditure, (b) calorie intake, (c) protein intake, (d) income inequality and (e) World Bank poverty indicator for coastal Bangladesh. Thick solid blue lines: simulated mean; Black dash lines: ±1.5 standard deviation (~85 percent); Shaded area: min-max simulated range; Red dots and diamonds: observations. Observations: a-c) BBS [56: Tables 4.4, 5.3, 5.4]; d) dots: rural inequality [59], diamonds: national inequality [60]; e) People living on less than US$1.90 a day [61].

Mean total expenditure matches the observations, both in terms of trend and magnitude. However, there are a wide range of simulated values (see grey area in Fig 4). Simulated mean calorie and protein intakes are also comparable with the rural-specific observations and the mean calorie intake is at the level of the Bangladeshi food poverty threshold (2122 kcal/capita/day). The GINI coefficient measures the income inequality of the population (i.e. the income distribution within the population). The coefficient ranges from 0% to 100%, with 0 representing perfect equality (i.e. everyone earns equally) and 100 representing perfect inequality (i.e. one person/household earns everything). Simulated mean inequality is higher than the national average observations (diamonds), but lower than the rural observations (dots). The simulated GINI coefficient follows the observed, increasing rural trend.

Finally, the World Bank’s national “People living on less than US$1.90 a day” indicator was compared against the simulation results. The simulated consumption expenditures were converted to USD and adjusted to the World Bank’s Purchase Power Parity (PPP) conversion factor before plotting. Initially this headcount indicator is significantly overestimated compared to the national average. As the simulation progressed, the poverty prevalence decreased substantially, and by 2005, it reached the magnitude of the observations. The initial deviation of simulated headcount poverty from the observations is surprising because the total expenditure (Fig 4A) matches the observations well, and expenditure and consumptions are closely related. Expenditure in HEAP is measured as Bangladeshi Taka (BDT) and the USD-based headcount poverty is calculated from this expenditure. Thus, the exchange rate and PPP conversion might be the source of deviation. Furthermore, the HIES observations are closer to the HEAP results perhaps indicating that the divergence in values is the result of comparing national (World Bank’s poverty index) with regional values (HIES and HEAP results). Finally, the well-being results of the 1990s might still be sensitive to the starting conditions, as discussed earlier. Overall, the model behaves as expected and reproduces national trends well.

### Creating a multi-dimensional poverty index

A multi-dimensional measure of poverty captures requires the aggregation of different dimensions such as calorie intake for health, child attendance in school for education and total value of owned assets for living standard. For HEAP we developed an index (MPI multi-dimensional poverty index) of these dimensions developed following the approach of Alkire et al [50] that characterises deprivation in health, education and living standards.

Following Alkire et al [50], a deprivation score was calculated for each of these proxies by comparing the simulated results with a benchmark value. A household is categorised as deprived if food consumed is less than the food poverty line of Bangladesh (2122 kcal capita^-1^ day^-1^), if it cannot pay for education, and if the total asset value (as defined by Alkire et al [50]) is smaller than the observed value for asset-deprived households in the seasonal household survey. The asset deprivation threshold in 2014 was identified by considering the value of the observed essential assets (e.g. cooking fuel, latrine type, table) in the seasonal household survey data [46]. 40,000 BDT was calculated as the asset deprivation threshold, and the value for 1990 (10,000 BDT) was back calculated from this 2014 value by using the World Bank's inflation rate for Bangladesh. The deprivation scores of the indicators are equally weighted in the final MPI. If the deprivation score is greater than zero, the household is considered poor in at least one dimension. Poverty is considered multidimensional, when the final deprivation score is more than 33% (i.e. this is our multidimensional poverty line). The calculated average MPI score for the household archetypes considers both the incidence and the intensity of poverty across all 653 unions.

## Results

To demonstrate the capability of HEAP, we selected eight household archetypes that show a range of different livelihood strategies with distinct long-term poverty trajectories. We use descriptive names (e.g. Small business with some farm labour) instead of the seasonality-based names (e.g. SmallBusiness—SmallBusiness–SmallBusiness (SLO)) for ease of reading, but the unique archetype IDs (e.g. 14) and the land ownership indicator (LL- practically landless, SLO–small land owner, LLO–large land owner) are always provided to allow identification and comparison. The selected groups are all landless or small land owners, because archetypes with large land holdings never fall below the poverty line in the simulation. The comparative characteristics of all household archetypes are listed in Table S1.1.

This chapter first characterises these selected archetypes but analysing their income and expenditure variations, and then discusses their long-term, seasonal poverty trajectories to present day and to 2030. Outputs for all archetypes are shown in Supplementary document 2.

### The effect of livelihood diversification on seasonal income variability

**Fig 5:**
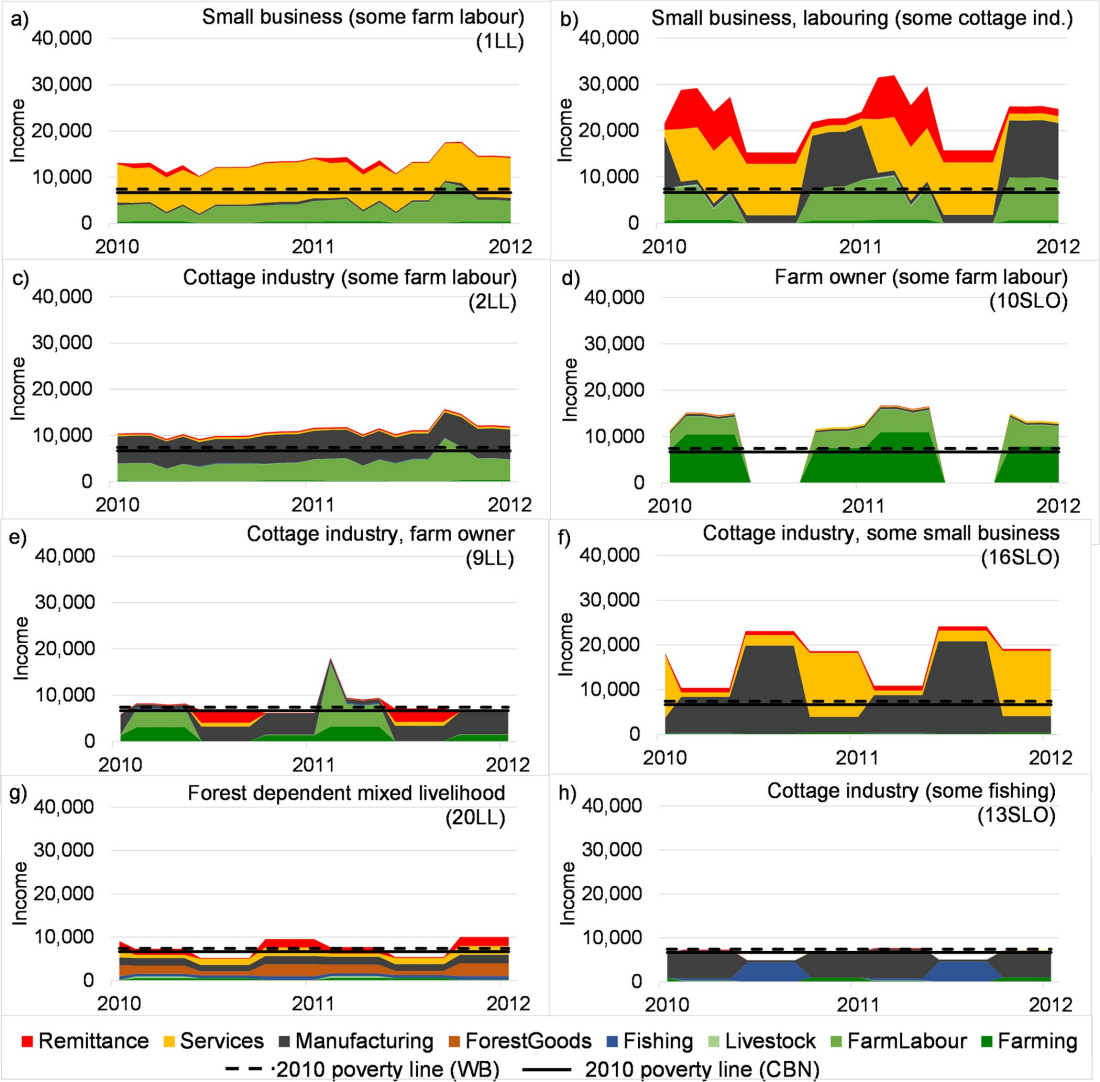
Two year sample of simulated intra- and inter-seasonal livelihood diversification and income-levels (thousand Bangladeshi Taka per month) of selected household archetypes of coastal Bangladesh. Consumption-based World Bank (WB) and Cost of Basic Needs (CBN) poverty lines for 2010 are also shown.

In this section, we visualise the level of seasonality incomes, the depth and duration of deprived seasons and the role of natural resource-based livelihoods in these dynamics (Fig 5, Fig S2.1). Some households predominantly relying on off-farm (non-natural resource-based) income sources (1LL, 2LL) gain the majority of their income from just one livelihood and use farm labour to earn additional income. They do not have any periods without income nor does the level of income fluctuate. Other non-natural resource-related households (7SLO, 16SLO) have large seasonal variations in livelihood income due to seasonal availability of off-farm employment. In this case, remittances can play a key role in providing additional income and ensuring a sustainable quality of life and thus well-being. Interestingly, these households own land, but do not use it productively, potentially because it is poor quality or they cannot afford the inputs required. Income in households with predominantly natural resource-based livelihoods has strong seasonality (10SLO, 9LL, 20LL, 13SLO). Agriculture is not viable in some seasons in parts of the study area (due to a lack of irrigation and/or seasonal soil salinization), leading to shortfalls in income (10SLO, 9LL). However, income levels can show high seasonality regardless of the use of natural resources (13SLO), levels of diversification or continuous presence of income sources throughout the year (20LL).

We include internationally comparable poverty lines on Fig 5 in order to show whether or not households would be considered poor, and to show that households are falling into and climbing out of poverty within one year. When converting the World Bank’s US$1.9 per capita per day poverty index to monthly values, in 2010, the poverty threshold corresponds to approximately 7,419 Bangladeshi Taka per household per month (BDT/household/month). When the cost of basic needs method of Bangladesh is used, the poverty threshold is 6,672 BDT/household/month (based on a rural mean of 1460 BDT/capita/month upper poverty line [56] and a household size of 4.57 [62]). Note that both poverty lines can vary over time due to variability of exchange rates and the cost of a basic set of food and non-food goods. Many income levels in Fig 5 only slightly exceed these poverty thresholds thus; they are at risk of falling into poverty. Certain household archetypes drop below the poverty line throughout the year for example, small landowners, those with forest dependent mixed livelihoods, and those reliant on cottage industries and open access resources to make ends meet.

### Long term trajectories of standard of living

**Fig 6:**
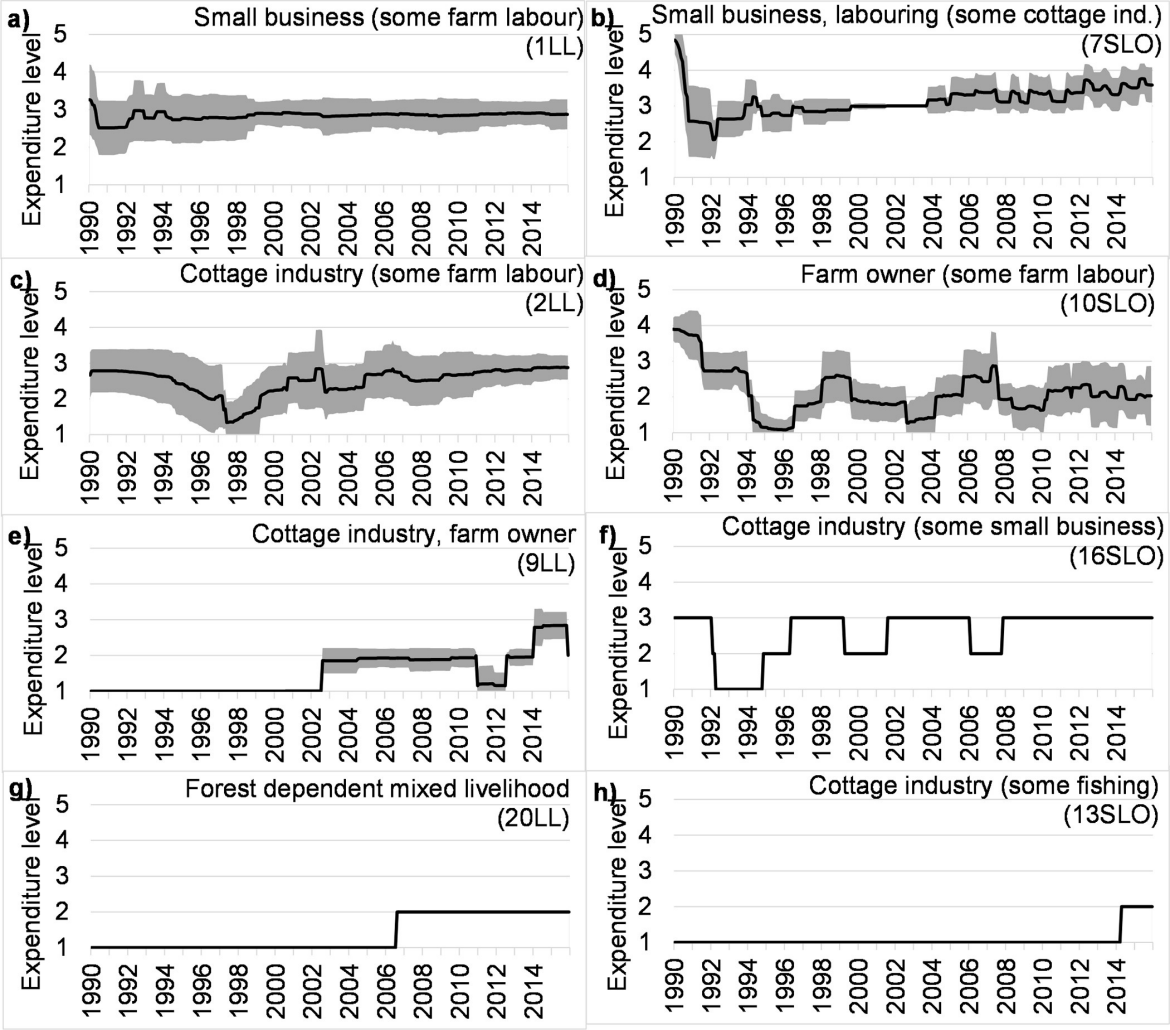
Simulated mean and ±1std range of expenditure level (1: poorest, 5: least poor) of selected household livelihood archetypes of coastal Bangladesh. Archetype ID is in brackets.

Income provides no information on the standard of living being reached by a household. Thus, an expenditure-based analysis of the quality of life is needed, to analyse which households can sustain or improve their standard of living over time (Fig 6; Fig S2.2).

By comparing income and expenditure levels (Figs 5 and 6), it is clear that income is not sufficient to explain expenditure. For example, the small business-based households (1LL) have income levels approximately 40 percent larger and much more constant levels than the cottage industry-based households (2LL), yet their expenditure levels are not much higher. Continuous income provides a safety net against the impact of sudden shocks. In comparison, without the continuous income, the farm owner household 10SLO struggles against a multi-year poverty cycle. In general, large seasonal variations in income generally result in low expenditure levels with little prospect of improvement (9LL, 16SLO, 20LL, 13SLO) regardless of whether the income is ecosystem or non-ecosystem-based. Shading on Fig 6 shows the spatial differences in simulated expenditure levels for each archetype. Household types with higher expenditure have more spatial variations in expenditure levels (i.e. wider shading) indicating diversity in the ways that households are applying coping mechanisms. The poorer households on the other hand all behave in the same way: taking high interest rate informal loans continuously to cover necessary expenditures. Thus, the ability to maintain the household’s standard of living strongly depends on both the level and stability of income, livelihood composition and the type of copying mechanism available or employed.

### Long term and seasonal fluctuations in multi-dimensional poverty trajectories

**Fig 7:**
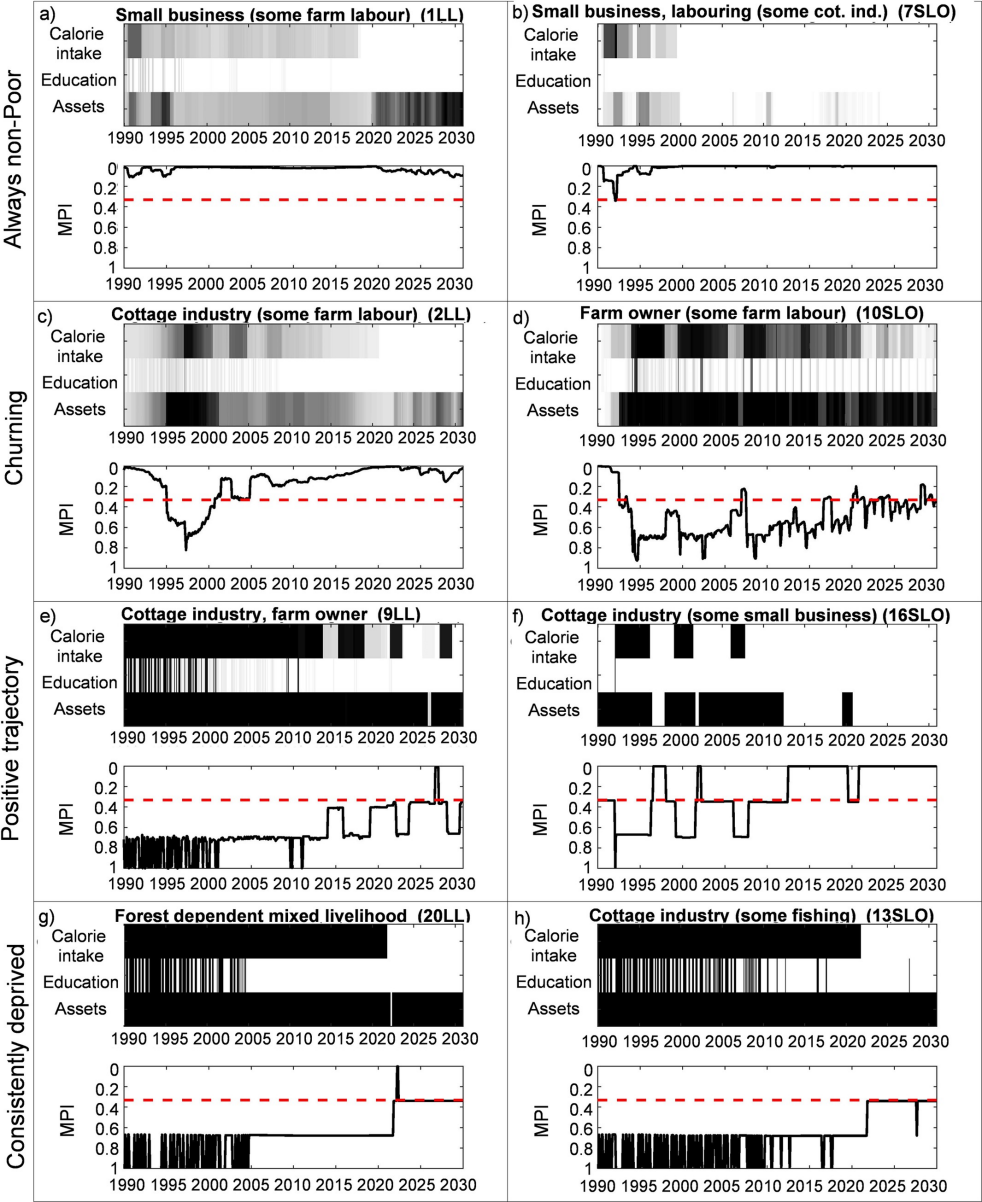
Poverty trajectories of selected household archetypes in coastal Bangladesh. The shade of the Calorie intake, Education and Assets indexes indicate the severity of the deprivation: the darker the more deprived. The red dashed line marks the multidimensional poverty line. MPI: Multidimensional Poverty Index.

The results (Fig 7, Fig S2.3) show that deprivation evolves over time for the simulated livelihood traits and these trajectories are different for different household archetypes. Four broad trajectories are observed: (1) Always non-poor, (2) Churning around the multidimensional poverty line, (3) Positive trajectories that may or may not cross the multidimensional poverty line, and (4) Consistently deprived on all measures. Large landowners did not show any deprivation on any of the multi-dimensional poverty indicators over the model run, regardless of income source. However, multi-dimensional poverty in small land owning and landless households varies.

In addition to owning sufficient land, three other factors seem to associate strongly with a lack of deprivation: (1) an income dominated by natural resources (both private and open access) in all seasons (e.g. 3SLO on Fig S2.3, also Fig S2.4e,4f); (2) access to income from business or salaried employment (e.g. 1LL, also Fig S2.4f); and (3) a permanent income source throughout the year (which may or may not be supplemented by alternative income sources (e.g. 8SLO, also Fig S2.4a). Dominantly fishing or farming households did not show deprivation at any point, regardless of size of landholdings (e.g. 3LL, 4LL, also Fig S2.4f). However, absolute levels of well-being differ with large landowners having much higher levels of income versus the other groups (see Figs S2.1, S2.3, also Fig S2.4d).

The results presented by the model support previous findings [7, 13] that off-farm employment alone is not sufficient to reduce poverty, and rather the quality of off-farm employment matters (i.e. the ability to generate high returns, see Fig S2.4f). Those households who accessed business opportunities or stable salaried employment (e.g. 1SLO, 2SLO), never showed deprivation on any poverty dimension of the MPI, although absolute income values were different just as for farmers. Generally, land ownership provides the capital that allows households to access high-end off farm opportunities such as small businesses and salaried employment [13, 63] but some landless households had also been able to access these opportunities (e.g. 1LL)–perhaps by selling land assets.

Some household archetypes are successful at using alternative (often non-farm) livelihoods to smooth income variations and thus maintain expenditure (7SLO on Figs 5 and 6). However, when considering all results (Fig S2.3) having one livelihood that provided an income across all seasons was more likely to be associated with a lack of deprivation (Fig S2.4a,4b). For example, 2LL has stable income sources as opposed to 9LL, who is forced to do multiple livelihoods when farming and farm labour are not possible (Fig 5). The lack of stability is clearly reflected on the MPI scores and showed severe deprivations for 9LL; whereas 2LL was better off with milder deprivations (Fig 7). Thus, seasonality in income type negatively affects multi-dimensional well-being (see ‘Mixed’ livelihoods on Fig S2.4e) consistent with the wider literature [14, 63].

There are large seasonal and inter-annual variations in households deprivation. The ability of households to pay for education shows the highest seasonal pattern, as households stop paying for education as a temporary response to a shortfall in income (20LL, 13SLO). Seasonality in deprivation (education, food) impacts not only immediate but longer term poverty trajectories due to the lost investment in human capital [64]. Such investment is currently not represented in HEAP and would require further research to understand how adaptation decisions are made and what other factors need to be considered to predict their long-term impact on well-being and poverty. Assets are also important well-being indicators as they indicate the long-term investment prospects [65, 66]. Our simulations show that most household types are asset-poor thus potentially limiting future positive well-being trajectories.

### Plausible future poverty trajectories

**Fig 8:**
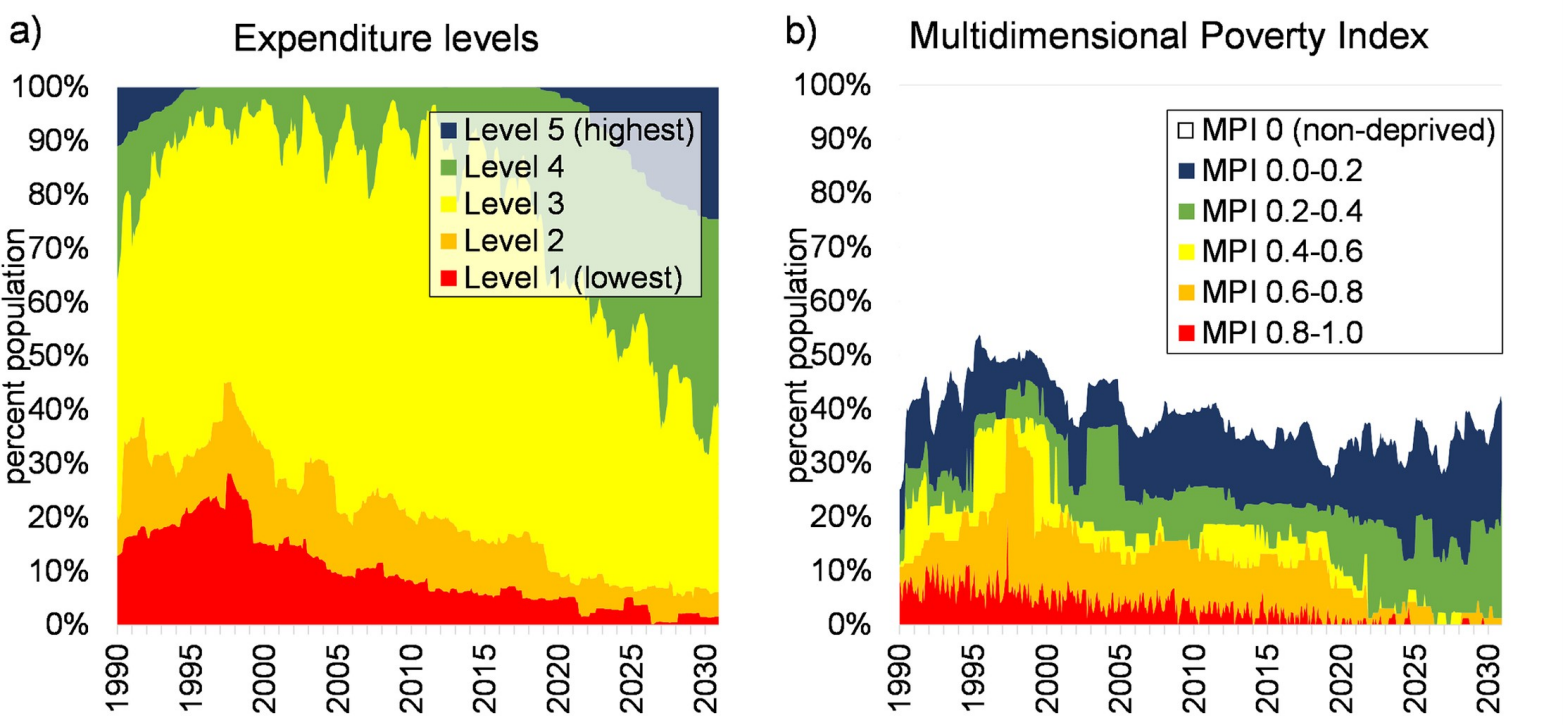
Distribution of monthly simulated expenditure level (a) and multidimensional poverty (b) in the study area considering all unions and archetypes.

The model results indicate a positive trend in poverty reduction towards 2030 (Fig 7). All simulated household archetypes experience an upward trajectory of expenditure, with less frequent multidimensional deprivation, but with different rates of increase. Complete poverty alleviation does not occur and many archetypes remain multi-dimensionally poor (e.g. 9LL and 13SLO, and all results on Fig S2.3). When the total population is considered, the well-being steeply increases after 2020 with the ‘extreme poor’ population (‘expenditure level 1’ on Fig 8A) gradually disappearing. Even though the household expenditure levels increase, household deprivation is not eradicated (Fig 8B). This is due to the fact that a better standard of living results in a higher social status within the community, and more coping mechanisms are used to maintain this. Debt levels increase, education expenditure is occasionally not paid, asset levels are low and food intake is sometimes suboptimal for some households. Fewer and fewer households experience extreme deprivation (MPI > 0.8 on Fig 8B) over time, and the number of least deprived households increases (MPI < 0.4) which is a positive sign of change.

Even though the economic trends are realistic, the HEAP results have to be considered with care as the model currently only assesses the development potential of household types with no dynamic adaptation, does not contain climate, environmental (e.g. cyclone) or family (e.g. illness) shocks, and sudden economic changes. It is difficult to judge if the results are too optimistic: stochasticity would make the results worse, but simulated household adaptations would make them better.

## Discussion

The results presented here show that natural resource use provides a safety net function and thus is associated with a lower incidence of poverty for households in coastal Bangladesh (Fig S2.4e), but only in those cases where these activities were practiced throughout the year (Fig S2.4e,4f). When the deficits are met using low return incomes sources (e.g. day labour in manufacturing jobs), the smoothing effect did little to help households stay out of poverty (e.g. 20LL). The results are consistent with a review of findings globally on the role of agriculture in rural development: that although livelihoods are diversified, agriculture appears to have a central role in enabling households to benefit from other rural activities [67].

Some of the most consistently deprived households, and those who show the highest seasonality in multi-dimensionality poverty, are those dependent on off-farm employment and cottage industries (e.g. 9LL, 13SLO, also Fig S2.4f). Many of these jobs support the farming and fishing sectors including through providing nets, seeds, fertilizers and other inputs. Therefore, it could be argued that seasonal stress is being transmitted from those with access to natural resources to those without access, something that has been documented in the case of large land owners transferring risk to workers on their farms [68].

Livelihood diversification is also the norm in other rural economies, for example in rural Africa, where non-farm income is associated with positive well-being [69]. Even though non-farm labour wages exceeds farm labour wages [19], quality non-farm opportunities require overcoming substantial investment and mobility barriers [69]. Such wealthier households can counteract seasonal effects, re-invest into farm-based livelihoods and profit from seasonality [63].

Our model structure and results are in line with the micro-scale conceptualisation of Barrett and Swallow [70], namely that households find themselves trapped at lower stable states from which they cannot easily transition. The reasons for being trapped in poverty include livelihood sources that are not continuous, insufficient assets, and inability to self-finance investments with high enough returns. Eliminating thresholds of poverty traps by providing, for example, training, salt/drought tolerant crops, guaranteed work, minimum wage, and subsidies are just as crucial as providing a safe and healthy environment for farm-based livelihoods [70, 71]. Other one-off investment programs targeting the poor, allowing them to invest in, for example, livestock rearing, are also promising [72]. Education and advantageous loan structures are also essential to help the chronic and transitory poor. HEAP can identify household types that may become trapped and test policy responses by altering the livelihood practices (e.g. crops, cropping patterns) and the financial and economic conditions (e.g. loan characteristics and labouring conditions).

However, poverty at the micro-scale is also influenced by processes at the regional and national scales that directly affect hazards, environmental quality and the economy for households themselves [70]. There are, therefore alternative levels for the most appropriate and effective development interventions [73, 74]. In the case of coastal Bangladesh, for example, infrastructure (i.e. polders) promote higher yields and reduce hazards, but also prevent natural sedimentation and soil fertilisation and increase relative sea level rise with longer-term adverse effects [75]. Dykes and triple-cropping might only benefit the wealthy [76] since they have access to land. Intensification of agriculture can cause soil salinization through irrigation [77], and conversion of land use can alter biodiversity and ecosystem functioning [28, 74]. Poverty also persists through wider inequalities such as environment quality, access to markets, and uneven imposition of regulation [78, 79]. Integrated assessments and models are useful to identify such trade-offs across space [80] and time and HEAP is being applied in this context.

HEAP characterises long term poverty trajectories for a range of livelihood diversification strategies in coastal Bangladesh, but the approach could be applied widely where survey data exist. The HEAP approach is novel in recognising that: (i) opportunities available to households to improve well-being and mitigate risk are not equally distributed; (ii) everyday decisions to smooth the seasonality of natural resource-based incomes affect poverty over longer timescales; and (iii) household decisions are motivated by social as well as economic factors. HEAP is based on an understanding of livelihood dynamics and hence is more realistic and credible than statistically based analysis. By doing so, it identifies household livelihood strategies that are more or less successful. Building on insights and established evidence on rural development and poverty and on detailed qualitative and quantitative primary data, HEAP can quantitatively investigate the dynamics of well-being and poverty at the household level. Once established and validated, such models have great potential to facilitate poverty analysis by providing consistent, long-term characterisation of the poverty dynamics of household archetypes at temporal resolution that could not be measured by surveys.

Individual and bespoke surveys of the economies and regions often use metrics for poverty and poverty reduction that are not directly comparable and problematic to measure consistently over time [81]. Our analytical strategy here is to use standard metrics and to approximate continuous, monthly poverty incidence. This generic approach is easily transferable to other rural developing country contexts where household characteristics (via household survey or HIES-type data) and trends of ecosystem service benefits and sectoral economics are both available. HEAP can simulate the effects of different policy interventions on households. For example, interest rates on loans, ability to accumulate cash savings, and preference for different coping strategies. HEAP also incorporates the role of community-level safety nets. In doing so, it provides a useful tool for policy makers on managing potential trade-offs between policies targeting the financial capital and poverty. Further improvements could be explored to make the approach more realistic, such as the inclusion of household adaptations beyond coping and social networks. In this analysis HEAP was used in a spatially limited mode, but it is designed to easily couple into integrated assessment frameworks, such as the Delta Dynamic Integrated Emulator Model (ΔDIEM) [82–85]. Such coupling realises the full potential of HEAP in assessing long-term household poverty trajectories across multiple plausible futures, including endogenous processes represented in other models, and enables the analysis of robust policy intervention strategies in the context of human-natural system dynamics.

## Conclusion

The aim of the paper was (1) to analyse the association between seasonal variation in livelihoods and income and poverty trajectories in coastal Bangladesh and (2) to assess the role of natural resource-based livelihoods in reducing the incidence of poverty over time. We achieved these by developing and applying the novel HEAP model incorporating key attributes of rural livelihoods at the household scale such as seasonality, intra-community inequality, social obligations and the coping strategies. The main results obtained from this study may be summarised as follows:

1. There is a complex interaction between poverty and land ownership and natural resource dependence over seasonal and annual timescales in coastal Bangladesh. Income security (continuous employment, multiple crops per year, etc.) rather than absolute income is most important for well-being and certainly for the avoidance of periods of poverty. This finding resonates with established findings in development economics on the acute risks of falling into poverty and their detrimental impact on long term development [9]. Even though a positive trend in well-being is simulated, complete poverty alleviation is unlikely before 2030 in coastal Bangladesh, when the progress towards the SDGs will be evaluated [80].
2. The poverty alleviation role of off-farm income types is significant, and underscore the importance of the stability and returns from off-farm employment when access to the benefits from natural resources are variable across seasons [13]. There are, however, limited opportunities for high value off-farm employment for the poorest households with limited capital in coastal Bangladesh. The analysis identifies people with small-scale enterprises as most likely to be poor and unable to escape poverty. Poverty outcomes of small landowners are highly variable, despite the role that land ownership plays in rural economies. This highlights the crucial nature of individual agency and other context-specific characteristics of households in creating distinct poverty trajectories.

## Supporting information

S1 File HEAP. (PDF)

S2 File All plots. (PDF)

S3 File Observed inputs. (XLSX)

S4 File Figure supporting data. (XLSX)
