# Supplementary material for: Modelling household well-being and poverty trajectories: An application to coastal Bangladesh
Source: PLoS One. 2020 Sep 4;15(9):e0238621. doi: 10.1371/journal.pone.0238621 (PMC7473571; doi:10.1371/journal.pone.0238621)
Supplement: S1 File — (PDF) [file pone.0238621.s001.pdf]

Supporting Information (1) for

Modelling household well-being and poverty trajectories: an application to coastal Bangladesh

Attila N. Lázár[1]*, Helen Adams[2], W. Neil Adger[3], Robert J. Nicholls[4]

[1] Department of Geography and Environmental Science, University of Southampton, Southampton, United Kingdom

[2] Department of Geography, King's College London, Strand Campus, London, United Kingdom

[3] Department of Geography, College of Life and Environmental Sciences, University of Exeter, Exeter, United Kingdom

[4] Tyndall Centre for Climate Change Research, University of East Anglia, Norwich, United Kingdom

*** Corresponding author:**

Email: a.lazar@soton.ac.uk

Contents of this file

Sections

S1. The purpose of the HEAP model 3
S2. Entities, state variables and scales in the HEAP model 4
S3. Process overview and scheduling within the HEAP model 6
S4. Design concepts 7
S4.1 Basic principles: general concepts and theories behind the calculations 7
S4.2 Emergence: the key outputs calculated from the behaviour of the households 9
S4.3 Adaptation: rules for decisions and adaptive traits of the households 11
S4.4 Objectives of the households during the simulation 15
S5. Submodels 16
S5.1: Loan calculation 16
S5.2: Land size calculation 17
S6. Initialization and calibration 18
S7. Input data 18
S8. References 23

Figures

Figure S1.1: Study area boundary and the Social-Ecological System (SES) classification of the unions in the year 2014 3
Figure S1.2: Component overview of the HEAP model 6
Figure S1.3: Sequencing of calculations 7
Figure S1.4: Observed relationship between mean food expenditure and mean calorie intake 10
Figure S1.5: Transitions between expenditure levels in the HEAP model 11

Tables

Table S1.1. Household archetypes based on the seasonally dominant livelihood and land size (based on the ESPA Deltas' household survey) 20
Table S1.2. Model parameter descriptions and values 21
Table S1.3: Expected economic changes in coastal Bangladesh between 2015 and 2030 (percent change) under a Business As Usual scenario 22

Supporting Information (Files uploaded separately)

S1 file: HEAP (S1_HEAP.pdf)
S2 file: All plots (S2_All_plots.pdf)
S3 file: Observed inputs (S3_Observed_inputs.xlsx)
S4 file: Figure supporting data (S4_Figure_Supporting_Data.xlsx)

The Household Economy And Poverty trajectory (HEAP) model is similar in nature to an agent-based model. Thus, the HEAP model is described here by using the ODD (Overview, Design concepts, Details) protocol developed for individual and agent based models [1].

S1. The purpose of the HEAP model

The project called 'Assessing Health, Livelihoods, Ecosystem Services and Poverty Alleviation in Populous Deltas' (ESPA Deltas) aimed to create a quantitative interdisciplinary assessment framework to study the relationship between climate/environmental and socio-economic change and livelihood, poverty and health in coastal Bangladesh, through the perspective of ecosystem services [2]. The model described here is the **Household Economy And Poverty trajectory (HEAP) model** which is the household component of the ESPA Deltas' integrated assessment framework called the Delta Dynamic Integrated Emulator Model (ΔDIEM). The purpose of the HEAP model is to analyse how natural resource-based livelihoods contribute to poverty alleviation at the household level, taking into account the constraints and opportunities of rural social relations and economies. Although the model is based on financial calculations, the outcomes of the model represent different dimensions of well-being, specifically expenditure, education and nutrition. The HEAP model, consistent with all components of the ΔDIEM model framework is coded and coupled in run-time within Matlab.

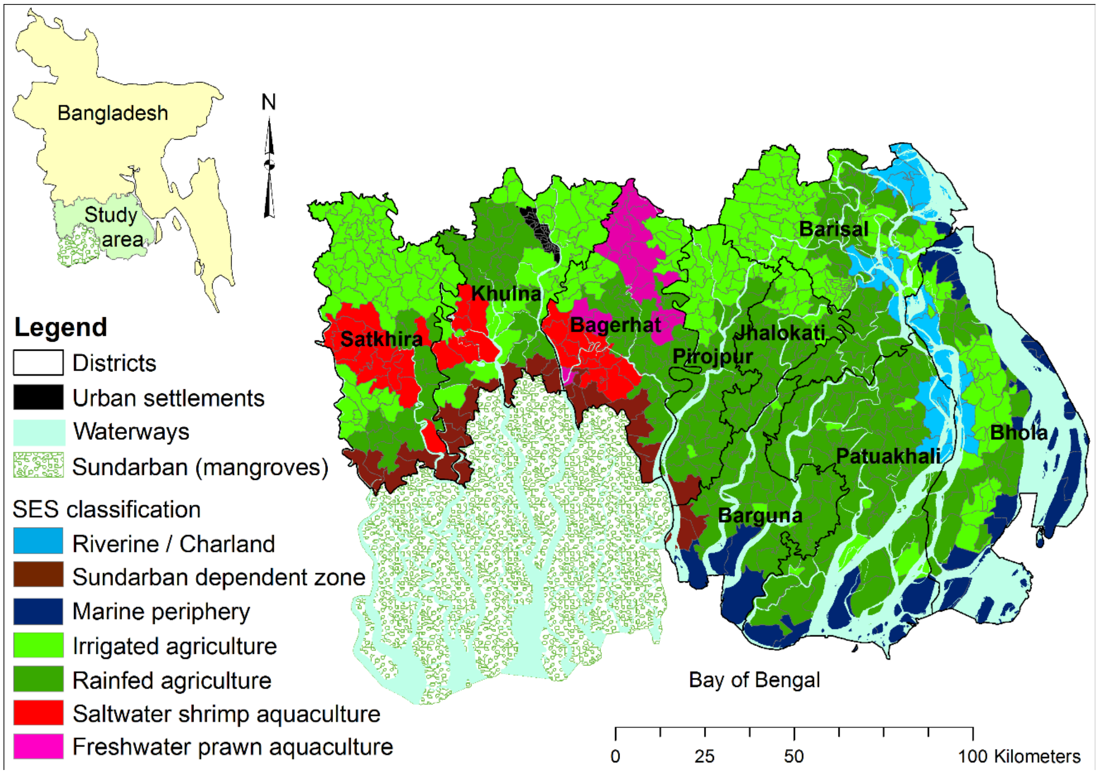

Figure S1.1: Study area boundary in Bangladesh and the Social-Ecological System (SES) classification of the Union Parishads in the year 2014

S2. Entities, state variables and scales in the HEAP model

The household component model comprises two hierarchical levels: unions and archetypal households. The study area (Figure S1.1) is subdivided into 653 administrative units, called Union Parishads (i.e. unions). Each union is classified into a Social-Ecological System (SES) based on its location and dominant land use composition [3]: (1) Coastal periphery (unions along the coast), (2) Riverine and Charland periphery (unions located mostly on the channels of the Lower Meghna), (3) Sunderbans dependent zone (unions adjacent to the Sunderbans mangrove forest), (4) Irrigated agriculture, (5) Rainfed agriculture, (6) Saltwater shrimp aquaculture and (7) Freshwater prawn aquaculture. The SES classification of each union can change over time together with the land cover and land use changes. Unions are characterized within the HEAP model by the following seven state variables:

(1) land use (ha),
(2) total population,
(3) distribution of household types within the union (represented by household archetypes, described later),
(4) agricultural yield (kg/ha and Bangladeshi Taka/kg or BDT/kg),
(5) fish catch (tons and BDT/kg),
(6) income from off-farm livelihoods (BDT/month) and
(7) labour opportunities within agriculture and aquaculture (labourer/ha).

Unions also have other state variables in the main ΔDIEM (e.g. soil salinity, etc.), but these are not reported here because they have already considered in the above state variables (e.g. agriculture yield) and thus they do not directly influence the HEAP model outcomes.

The HEAP model distinguishes 36 archetypal households (Table S1.1), based on the ESPA Deltas' household survey dataset [4]. Archetypal households are based on the levels of livelihood diversification across three seasons and the degree of land ownership; these variables were chosen to capture differences in dependence on natural resources. Within each union, the composition of households (i.e. percent presence of each of the archetypes) are defined by the SES classification of the union with initial values based on the household survey data. For example, forest good collector archetypes are only present in the Sunderbans dependent zone SES. In subsequent year, the composition of households within a SES depends on the changes in household land sizes (governed by demographic and land cover changes) and the land protection threshold (i.e. the minimum sustainable land size for farming). Each household archetype is assumed to be representative of an average household of its type within the specific union. Households are characterized by the following 18 state variables:

(1) livelihood income (BDT/month),
(2) livelihood-related expenditures (BDT/month),
(3) food related expenditures (BDT/month),
(4) essential household expenditures (cooking fuel, heating, etc.; BDT/month),
(5) non-essential household expenditures (clothing, ornaments, etc.; BDT/month),
(6) education expenditures (BDT/month),

(7) health-related expenditures (BDT/month),
(8) house improvement expenditures (repair, building; BDT/month),
(9) social expenditures (wedding, funeral, etc.; BDT/month),
(10) repayment status of loans (formal or informal, number, months and amount remaining to repay) and their monthly expenditures (BDT/month),
(11) relative expenditure level (no unit; five groups),
(12) expenditure sub-level (no unit; six groups),
(13) zero expenditure status (household is surviving without formal expenditures; yes/no),
(14) total cash savings (BDT),
(15) total value of assets (BDT),
(16) household coping strategies employed (no unit),
(17) land size (ha), and
(18) number of households in the union belonging to that particular household archetype (number)

The spatial scale of the HEAP model is one administrative unit (the union). Unions are the smallest administrative and local council units in Bangladesh having an average surface area of 26 km^2 and containing approximately 9 villages and 21,000 people. Households within each union are not spatially differentiated.

The temporal scale of the HEAP model calculations is monthly; except three household state variables that are computed on an annual timestep: (1) the number of households, (2) land size, (3) dependency ratio. The exceptions for the union state variables are: (1) land cover, (2) population size and (3) household composition that are also computed with an annual timestep, and land use which is calculated on a seasonal timestep (every four months). Finally, the biophysical inputs (e.g. crop yields) have a daily time step. The HEAP model harmonises the temporal scales by continuously aggregating and interpolating between the timescales to create monthly inputs and thus monthly calculation steps. The way in which these model states are incorporated into model process is described in the following section.

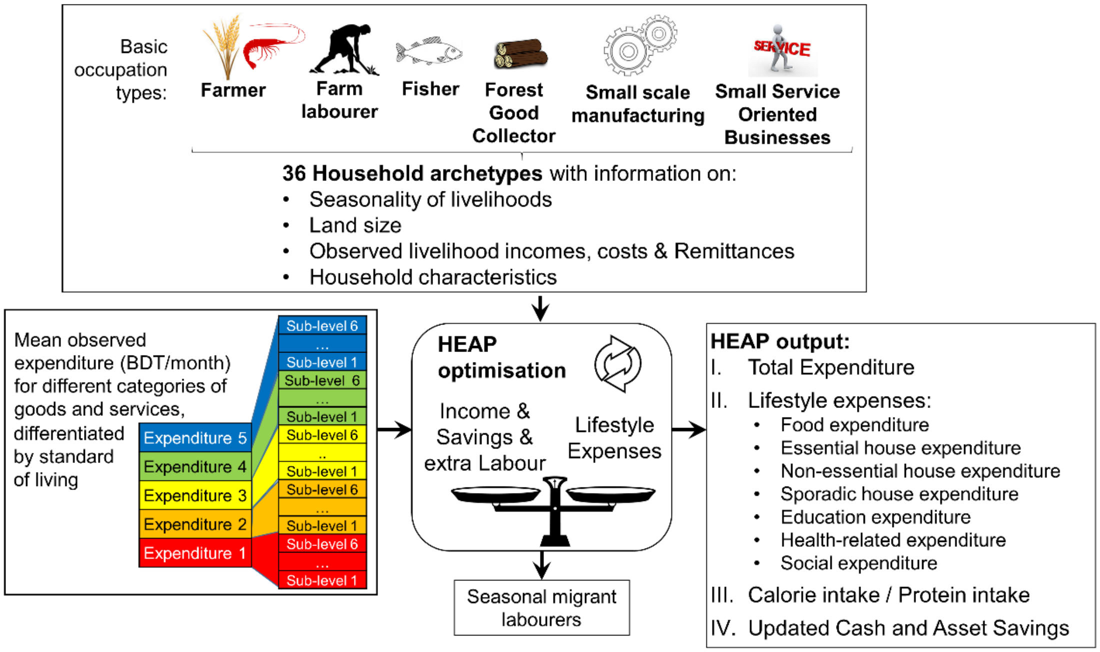

Figure S1.2: Component overview of the HEAP model

S3. Process overview and scheduling within the HEAP model

In brief, the HEAP model simulates the economic status (i.e. incomes and expenditures) of archetypal households by month and administrative unit (union), including the use of remittances and loans (Figure S1.2). During the calculations, households are assigned to an expenditure level based on affordability defined initially by values from the Household Income Expenditure Survey (HIES) and the ESPA Deltas' household survey dataset. A fundamental process in the model is that households work to maintain quality of life (smooth expenditure levels) despite falling income levels. The model uses inputs of natural resource productivity (agriculture, aquaculture and fisheries – accounting for climate and other environmental change), socio-economics (off-farm livelihoods, economic changes, land cover and land use changes) and demographics. Households can move between expenditure levels, based on their ability to meet a set of expenditures, which are expected at that income and savings level. The model analyses each union separately. Therefore, interaction of archetypes across unions is not considered. The sequencing of the calculation is illustrated on Figure S1.3 and Figure S1.5.

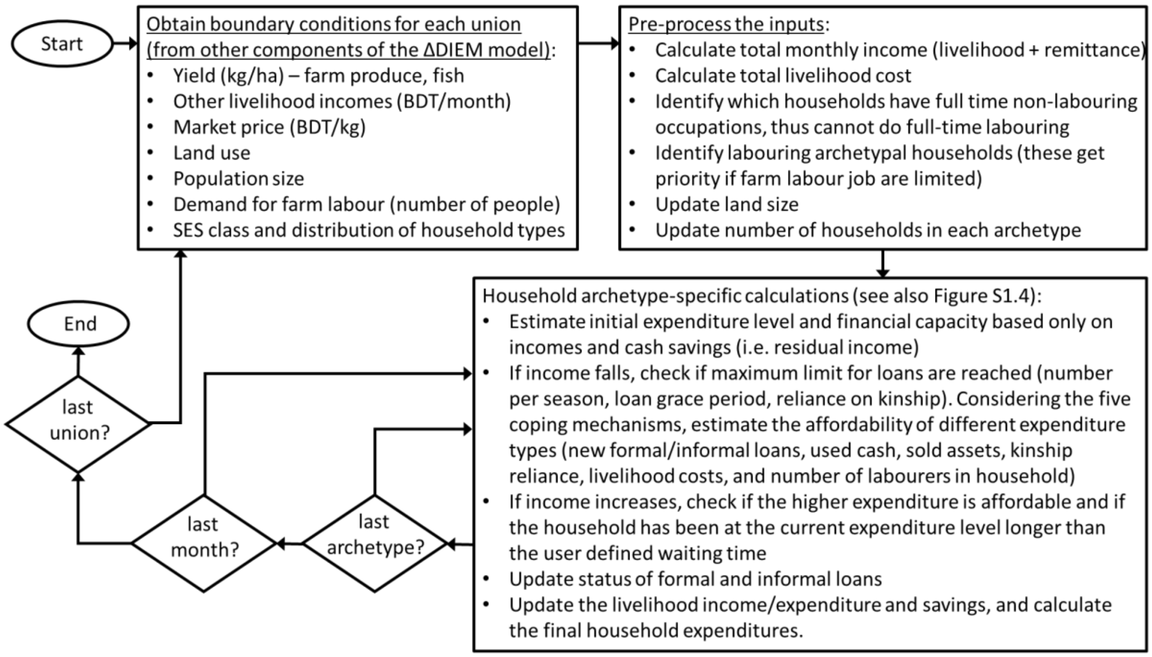

Figure S1.3: Sequencing of calculations

S4. Design concepts

The following sections details eleven design aspects of the HEAP model following the aforementioned ODD protocol recommendations.

S4.1 Basic principles: general concepts and theories behind the calculations

Poverty is a multi-dimensional concept [5]. The HEAP model focuses on the material and health-based dimensions (and measures) of poverty. Material poverty is expressed through household expenditure levels. Expenditure levels were used over other indicators of material poverty (such as income or assets) because they (i) indicate whether minimum basic needs are being met, (ii) allow the model to take into account the smoothing (or not) effect of loans and savings on fluctuations in income, and (iii) to see whether households are able to survive periods with no income at all [6].

The unit of analysis is the household archetype. Household archetypes are created based on observed seasonality of livelihoods and land ownership. Since decisions on risk spreading and income diversification tend to be made at the household level in poor rural settings, this is the most appropriate level of analysis [7]. Intra-household inequalities, for example between men and women, are thus not considered in the current version of the HEAP model. Households are categorised by their seasonal livelihood strategies in order to account for the relative success of different seasonal strategies. Seasonal poverty is widespread in agrarian economies, driven by weather and crop failure, and indirect effects on seasonal demand for labour. Livelihood diversification is a well-established strategy for households to attempt to reduce the negative impacts of such effects.

Decisions are made at the level of the household, and can include the migration of a family member to another labour market.

The ‘poor’ are not monolithic and different groups experience poverty in different ways [8]. Many households in rural Bangladesh move regularly into and out of poverty [8, 9]. Poverty can be chronic or transitory and households in rural areas are affected by seasonal patterns of work availability and natural resource productivity [10]. Poverty is also influenced by land ownership (represented in the HEAP model with land size) and status within the community (represented with relative expenditure level). Inequality contributes to how people experience poverty, not only because of the corrosive impacts on perceived unfairness in society [11], but also because inequality drives processes of vulnerability to shocks, seasonality and extreme events, as well as poor health [12, 13], and hence constrains options and opportunities. Households act to maintain their social status and meet social norms [14] even if this requires undermining strategies such as debt. Coping strategies vary with expenditure level [9, 10, 14]. We represent this with the settings of coping priorities and the rule on uptake of labouring jobs (see section 4.3).

The main framework of the model is based on a simple household economic mass-balance calculation (Equation 1) that matches income with expenditure:

$$\begin{aligned} \textit{Residual Income} &= \textit{Total Income} + \textit{Total Savings} - \textit{Fixed Expenditures} = \\ &= \textit{Total Livelihood Income} + \textit{Total Savings} + \textit{Remittances} + \\ &\quad \textit{Loan Income} - \textit{Livelihood expenditures} - \textit{Loan expenditure} \quad (1) \end{aligned}$$

Total Income includes *Livelihood Income* (farming, fishing, off-farm, etc.), *Remittance*, and if a loan was taken, the amount of the *Loan*. *Total Savings* include *Cash* and (productive and non-productive) *Asset* savings. *Fixed Expenditures* refer to expenses that has to be paid such as *Livelihood Expenditure* (e.g. seeds, labour hire) and *Loan Expenditure* (if an earlier loan has not yet paid back in full). The *Residual Income* can be used to pay other expenses around the household including: (i) food, (ii) day-to-day expenses (e.g. cooking fuel, electricity), (iii) non-essential expenditure (e.g. clothing, furniture), (iv) sporadic expenses (e.g. house repairs, productive assets), (v) education, (vi) health and (vii) social expenditures (e.g. marriages and funerals).

Household expenses are prioritised based on necessity. In a time of income shortage households prioritise food expenses, then other day-to-day household expenditures such as cooking fuel or electricity. If both these expenditures have been met then the model allows households to purchase less immediately necessary items such as clothing and furniture, and finally sporadic expenditures such as house repairs, expenditures associated with marriages and funerals. Education and health-related expenses are expressed as separate expense groups. While households may prioritise education of children depending on the attitudes of parents [15], in this preliminary modelling exercise, it is assumed that food expenditure, essential house expenditures, loans and livelihood expenditures have to be met before education is paid for the children. The timing of health-related expenses cannot be predicted and out-of-pocket payments for healthcare can push households into a negative poverty trajectory [16]. Due to the infrequent, random and variable nature of healthcare expenses, the model cannot simulate them

realistically. The HEAP model currently assumes that if the household has the financial capacity, a flat monthly fee is paid, similar to a health insurance. This flat rate is calculated from the ESPA Deltas' household survey dataset (i.e. the average amount of money households of different income levels expend on healthcare) and is only paid if all other expenses are met. Active (official and informal) loans are assumed to be as important to pay as the essential house expenditures such as cooking fuel and electricity.

Finally, *Remittance* from migrant household members is an important way for households in rural locations to spread risk and to invest in their livelihood [7, 17]. Thus, remittances from household members who have migrated to alternative labour markets are also included in the household economic balance (based on observed values from the ESPA Deltas' household survey dataset).

S4.2 Emergence: the key outputs calculated from the behaviour of the households

The model outcome is the relative expenditure level of the simulated household that is somewhat analogous to well-being. Expenditure was used over other indicators of material poverty (such as income or assets) because they (i) indicate whether minimum basic needs are being met, (ii) allow the model to consider the effect of loans and savings on fluctuations in income, and (iii) indicate if households are able to survive periods with no income [6]. Thus, the HEAP model not only allows households to move between expenditure categories at each time step, but also enables coping mechanisms to maintain expenditure levels in income-poor periods.

There are five of these relative expenditure levels in the HEAP model. These ranges are not quintiles (i.e. do not comprise equal numbers of observations). Rather, as our data is log-normally distributed, they are defined based on the probability distribution of the log transformed total household expenditure. Using the log transformed total household expenditure also ensures that the poorest households, of particular interest to this study, can be clearly identified. The expenditure levels vary with time in the HEAP model. The temporal change of expenditure levels are 'measured' using the same analysis on the HIES 1991, 1995/96, 2010 and the ESPA Deltas' household survey datasets, plus linear interpolation to fill the historic gaps in the time series.

Based on the established correlations between the different measures of poverty and the financial capacity of the household, other poverty indicators are also calculated: calorie and protein intake (kcal/capita/day, gram/capita/day, respectively), plus the ability to pay for education and healthcare. Based on the HIES and the ESPA Deltas' household survey datasets, we link food expenditure with a specific food basket and food quality, so, food expenditure is directly related to the calorie and protein intake of the household. These observations show that the food expenditure – calorie intake relationship is non-linear (Figure S1.4). Own-produced food is an important factor in rural food security. Thus, the model also considers what percentage of the crop is sold and what percentage is kept by the household for their own consumption (see 'Sold/Grown Crop Ratio' in Table S2). This ratio is considered in the crop income calculation, and is also considered in the calorie intake look-up table.

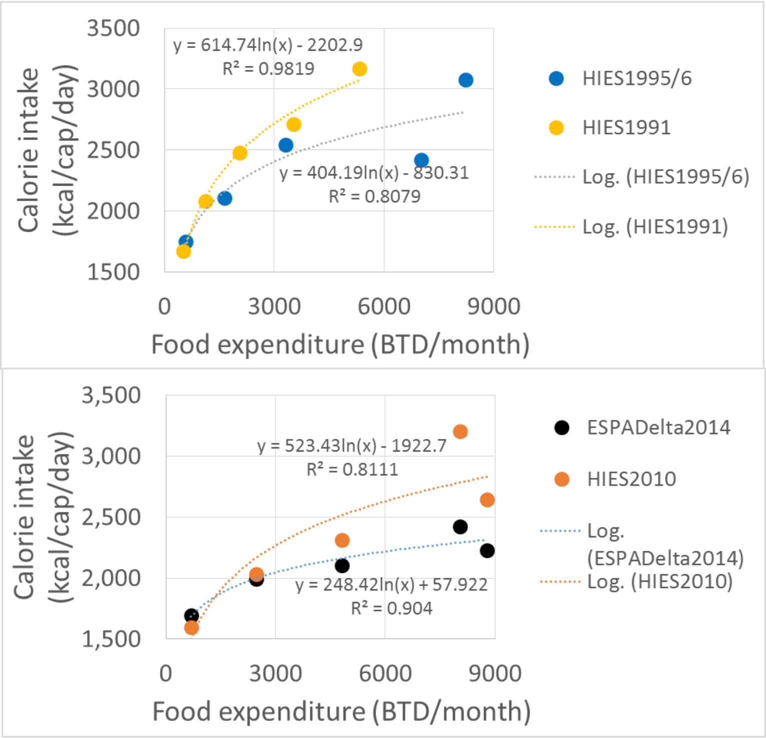

Figure S1.4: Observed relationship between mean food expenditure and mean calorie intake

Taking up labouring activities is an additional coping mechanisms in the HEAP model (see next section). By considering the available labour job opportunities (based on the agriculture practices and thus demand for labourers) and the financial conditions of the households, the HEAP model approximates both the local and seasonal migrant day-labour workforce at each timestep and for each union:

$$LabDem = \sum_{CP} \sum_{crop} \left(LabNeed_{CP,crop} * Area_{CP,crop} \right) \quad (2)$$

$$Workforce_{total} = \frac{LabDem}{4.28 * 6} \quad (3)$$

$$Workforce_{migrant} = Workforce_{total} - Workforce_{local} \quad (4)$$

where *LabDem* (i.e. labourer demand; personday) is the total days in a month required to cultivate the agriculture and aquaculture fields within a specific union, *LabNeed* (i.e. labour need; persondays/hectare) is the labour requirement of a specific crop in a specific cropping pattern (CP), Area (ha) the cultivated area assigned to a specific crop in the specific cropping pattern. The $Workforce_{total}$ is the number people required to do all agriculture labour activities considering 4.28 weeks per month (=30/7) and 6 working days. The $Workforce_{local}$ (number of people) is calculated as part of the coping strategies calculation, and the $Workforce_{migrant}$ (number of people) is simply the difference between the total demand for labourers and the local labourers.

The total value of assets (Assets; Bangladeshi Taka: BDT) at month '*t*' are calculated as:

$$Assets_t = Assets_{t-1} + Exp_{nonessentialHouse,t} + Exp_{sporadicHouse,t} - AssetLoss_t \quad (5)$$

where Exp_{xxx} (BDT) is the expenditure used to buy assets, and *AssetLoss* (BDT) is the sold assets to augment the cash flow of the household to cover everyday expenditures or cover loan expenditures when no other options are available.

Finally, by considering the level of expenditures (e.g. education), food intake and assets of the households, headcount poverty indicators and multi-dimensional poverty indices can be also approximated both at household archetype, union, district and study area levels such as the World Bank's £1.25 and $1.90 consumption headcount poverty index, food insecurity, hunger periods and multi-dimensional poverty index.

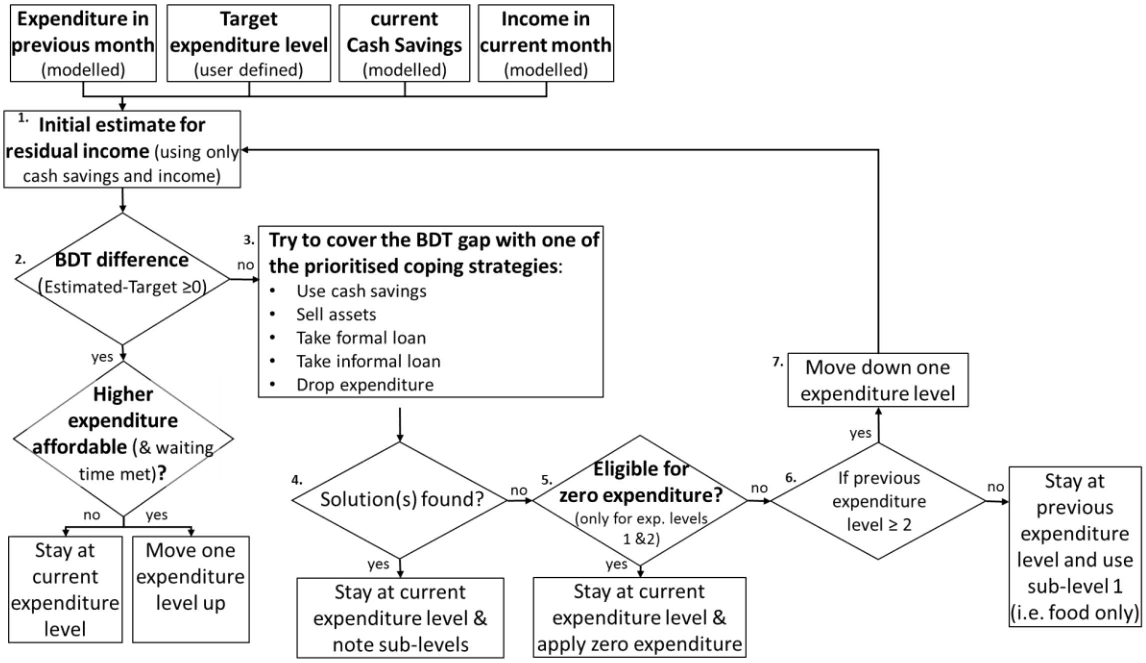

Figure S1.5: Transitions between expenditure levels in the HEAP model

S4.3 Adaptation: rules for decisions and adaptive traits of the households

The HEAP model estimates the actual expenditure that is affordable from the residual income (i.e. financial capacity) for the household at each time step. This calculation includes user-defined rules that determine whether the household spends within its means (i.e. reduces expenditure to match drop in income) or attempts to maintain the same level of expenditure (using coping strategies).

Ascending households

The HEAP model compares incomes, savings and expected expenditures. If the household is not stressed financially (i.e. all necessary expenses can be paid from income and cash savings), the model assesses whether the simulated household has both the financial capacity and is eligible to move up one expenditure level (Figure S1.5). Eligibility is based on a user defined waiting time (Table S1.2) during which the household has to be able to pay the maximum expenditure level of the current expenditure level. This ensures that the household does not increase its total expenditure too early, resulting in a potential need for extra loans and unsustainable transitions between expenditure levels. Thus, the HEAP model assumes that the household accumulates cash and assets before it significantly increases in expenditure.

Descending Households

If there is a deficit in the household economics, the HEAP model applies one or more coping strategies (Figure S1.5). For the poorest households, zero expenditure is also allowed, when the household entirely depends on stored food reserves, charitable assistance from relatives and neighbours, begging or access to government social safety net programmes. If none of the considered strategies maintain the current expenditure level, the household has to reduce its expenses and thus move down one expenditure category. If the household is already at the lowest expenditure level, the model allows the household to pay for the minimum food expenditure and household cash savings become negative (i.e. the household borrows money from someone with no-interest). The ESPA Deltas' household survey dataset shows that households, in general, drop a maximum of two to three expenditure levels within a season [4]. To be consistent, in the HEAP model households can reduce their expenditure levels by one category per month. These households can be considered 'descending poor' [8] and without intervention are destitute and socially marginalised [8, 18].

Coping strategies

Households prefer certain coping strategies to others based on the degree to which it is possible to bounce back and recuperate. Spending cash savings is always the preferred choice due to its easy mobilisation. Loans are preferred to selling assets [19], because the sale of assets often leads to spirals into chronic poverty [20]. Informal loans are preferred to formal loans [19], because they are easier to access with less bureaucracy or fewer conditions. However, coping strategy priorities of the households can be defined by the user for each expenditure level (Table S1.2). Furthermore, the user can define the conditions of cash spending, selling assets, and the conditions of both official and informal loans.

In case of the 'drop expenditure' option, the HEAP model allows households to prioritise certain expenditures over others. Thus, to remain at the current expenditure level, when income falls short of necessary expenses, households reduce expenses in the following order:

1. Sporadic house expenses, Health-related expenses, Social expenses
2. Non-essential house expenses
3. Education-related expenses
4. Livelihood expenditures
5. Loan expenditures (if any), Essential house expenditures
6. Food expenditure

This reduction does not mean that the household stops all payments of these types (unless it is in the lowest expenditure level already); rather the paid expense is one expenditure level lower than what the current expenditure level would require. This reduction is also true for the direct livelihood expenditures (e.g. not paying for fertiliser). If this reduction in livelihood expenditures is necessary, the income from the livelihood type is adjusted to the affordable level of direct livelihood expenditure.

This livelihood income adjustment is done by using a logarithmic function (equation 6), where the minimum income (when payment completely stops) is defined by the user (Table S1.2: Income drop parameter). As a result of the logarithmic function income is only reduced slightly when the drop in expenditure is small, and the income does not become zero even if the household pays no livelihood expenditure. This is because crops still grow (with a lower yield) and some fish can be caught even without financial investments. This income penalty is instantaneous (i.e. does not consider for example time to the next harvest) because the ΔDIEM model, with its current structure, cannot estimate such changes for the non-ES-based livelihoods. This income penalty does not affect remittances or income from manual labour.

$$IncPenalty = \begin{cases} \dfrac{100 - MaxIncDrop}{2} * \log_{10}\left(DropInLivExp\right) + IncomeDrop & if \;\; DropInLivExp > 0 \\ IncomeDrop & otherwise \end{cases} \quad (6)$$

where *IncPenalty* (i.e. income penalty; percent) is the reduction of livelihood income if livelihood expenditures are not fully paid; *MaxIncDrop* (i.e. maximum income drop; percent) is a user defined value for the maximum reduction of livelihood income if no livelihood expenditures are paid; the ‘2’ in the above equation is the maximum possible value (i.e. log10(100) = 2); and *DropInLivExp* (i.e. drop in livelihood expenditure; percent) is the level of reduction in livelihood expenditure payment.

This reduction in livelihood expenditure is conceptualised as dependent on the decline in the total expenditure of the household. The level of reduction is the same as the level of reduction in the household expenses. If the household pays the expenses one level lower than the current expenditure level requires, the same percentage reduction is also reflected in the livelihood expenditures (equation 7). Please note that asset-related expenses (non-essential house expenditures and sporadic house expenditures) are not paid if expenditure declines; thus they only appear in the denominator:

$$DropInLivExp = \frac{Exp_{reduced}}{Exp_{total}} * 100 \quad (7)$$

, where

$$Exp_{reduced} = Exp_{essentialHouse,WL-1} + Exp_{education,WL-1} + Exp_{health,WL-1} + Exp_{social,WL-1}$$

$$Exp_{total} = Exp_{essentialHouse,WL} + Exp_{education,WL} + Exp_{health,WL} + Exp_{social,WL} + Exp_{nonEssentialHouse,WL} + Exp_{sporadicHouse,WL}$$

and Exp_{xxx} represents the different expense types, *WL* denotes the actual expenditure level at which the expenses should be paid, and *WL-1* marks the expenditure level just below the current.

Other coping strategies that the households can utilise include relying on friends and family members (i.e. zero expenditure levels for a user defined number of months) or using the loan grace period to repay loans (i.e. postpone the payment for a user defined number of months). In the HEAP model, these coping options do not have increased expenditure consequences; rather they provide a temporary relief to the household for a user defined number of months. The model keeps track of the used number of 'relief' months for the loans and the number months per year in the case of kinship-supported zero expenditure coping.

Household labour requirements

Farm labour is one of the livelihood types in the HEAP model, but this is not an input scenario, rather dynamically calculated, consistent with the often ad-hoc and needs-based use of day labour in rural households. The nature of the relationship of households to day labour is initially defined by the ESPA Deltas' household survey dataset. If the household archetype was observed to engage in regular or occasional farm labouring, the model considers this as an option for 'normal' or everyday income generation. If the labouring was observed as regular (i.e. the dominant livelihood type in that season), it is assumed that a minimum of one household member is engaged in day-labouring, but the number of labourers can be increased if the financial condition of the household is stressed and thus they need ore income. Inactive household members such as the elderly and children are considered through the dependency ratio state variable. Elderly and children are only allowed to do labour work if the household is at the lowest expenditure level and they do not pay education expenses If the dominant household livelihood type is not farm labour, the maximum number of household members eligible for additional labouring activities is reduced by one (i.e. at least one household member has to engage in other activities full time).

Labour job opportunities are estimated based on the total farm size and cropping practices of the union in question (see equation 2). The HEAP model allocate labour jobs first to the archetypes that are regular labourers (i.e. dominant livelihood type). Thus, in any simulation, it is possible that a certain archetype cannot take labour jobs due to the imbalance of demand and supply. Seasonal labour migrants are not considered explicitly in the model; rather the model fills surplus labour requirements (i.e. that were not taken by the local workforce) by seasonal migrants from outside the simulation (see equation 4).

S4.4 Objectives of the households during the simulation

The overall objective of the household is to increase their expenditure level. The households aim to move up from lower expenditure levels to higher ones as soon as possible (see *WaitingTimeUP* in Table S1.2). They also aim to achieve and maintain a target expenditure level that ensures that their basic needs and aspirations are met (e.g. education, clothing, etc.; see *TargetExpLevel* in Table S1.2). The financial capacity of the household to meet the target expenditure works the opposite way of the expenditure reduction levels, describing what the household has enough money for. Thus, financial capacity has the same elements, but is listed in the opposite order:

1. Food expenditure
2. Loan expenditures (if any), Essential house expenditures
3. Livelihood expenditures
4. Education-related expenses
5. Non-essential house expenses
6. Sporadic house expenses, Health-related expenses, Social expenses

The six categories are the same at every expenditure level (see also Expenditure 1 to 6 on Figure S1.2); however, the amount of money that they spend on these are different at each expenditure level (see Typical expenditure levels worksheet S3_Observed_inputs.xlsx). The household can choose which of these expenditures they are willing to sacrifice when income falls to maintain their status in their community. This means that food expenditure is the most essential to pay and home improvement, health and social expenses represent the least essential payments. At each expenditure level, the household decides what is affordable to pay (starting from food and considering 'other' expenses at last). *TargetExpLevel* is one of these six levels, defined by the user that the household aims to achieve and maintain. If this level of expenditure at the specific expenditure level is reached, no further coping strategies are used by the household.

Coping strategies of the households are changed over time in line with their expenditure levels (see *CopingStrategies* in Table S1.2). Households are not allowed to change their livelihood compositions (although this is a potential future enhancement of the model). Thus, the HEAP model quantifies the long-term suitability and well-being prospect of each archetypal livelihood composition.

Households do not make predictions about their long-term prospects or the likely future effects of their chosen coping strategies (e.g. sell assets). They react to the present circumstances. They also optimise their number of labouring members to in order to reduce/eliminate the necessary damaging coping mechanisms. We note that the affordable expenditure of the household at time *t* is given by:

$$f(\exp, t) = \{(ExpL(t),\ finCap(t)) \mid inc(t), saving(t-1), m(t), cs(t)\} \tag{8}$$

where the expenditure level $exp \in E$, where *E* is a set of five expenditure levels, *ExpL* is the current expenditure level, $finCap \in$ A is the financial capacity level with a set of six levels, *inc* is the income, *savings* is the total value of the cash and non-productive asset savings, the number of labouring members of the household $m \in M$, and *cs* is the selected coping strategy $\in CS$. The problem is to find a solution that:

(1) minimises the number of members engaging in labour work (i.e. maximising the deviation between the total members and the labouring members – equation 9),
(2) minimises the number of coping strategies (i.e. maximising the deviation – equation 10), but
(3) maximises the financial capacity level while maintaining a minimum net savings (i.e. at least 10% of income remains after paying all expenditures and expenses – equation 11):

$$\max\{M - m(t), 0\} \tag{9}$$

$$\max\{CS - cs(t), 0\} \tag{10}$$

$$inc(t) - f(\exp, t) > 0.1 * inc(t) \quad \text{i.e.} \quad \max\{0.9 * inc(t) - f(\exp, t), 0\} \tag{11}$$

The three objectives stated in Equations 9 to 11 are combined into a single objective function during the model run and solved heuristically:

$$\max \sum_{m \in M} \sum_{cs \in CS} \max\{0.9 * inc(t) - f(\exp, t), 0\} \tag{12}$$

where *m* is the number of members engaging in labouring activities, *M* is the active household size (household size minus dependents), *cs* is the applied number of coping strategies, *CS* is the available coping strategies, *inc* is the total income, *exp* is the total expenditure, *t* is timestep ‘t’.

If a solution that meet all three objectives is not possible, the second best solution is used (i.e. paying all expenses, but no net saving in the current timestep). If still no solution is found, the HEAP model uses the case with the maximum allowed labourer members and the maximum available coping strategies.

S5. Submodels

S5.1 Loan calculation

The model differentiates two loan types: official and informal loans. Loan repayment comprise capital repayment and interest payment. The interest is calculated based on the capital that has to be repaid. When the loan is taken, the first payment is due the following month. The monthly instalment is calculated using Equation 13 for both informal official loans that are given for 12 months or less (see amount, length, APR in Table S1.2):

$$MonthlyPayment = \frac{LoanAmount}{LoanLength} * \left(1 + \frac{APR}{100}\right) \tag{13}$$

Where *MonthlyPayment* (BDT/month) is the monthly expenditures associated with the loan, *LoanAmount* (BDT) is the amount of the initially borrowed money, *LoanLength* (months) is the time that is given to fully repay the initial amount plus the interest, and the APR (%) is the interest rate.

If the official loan is given for more than 12 months, the borrowed capital is updated annually by deducting the capital that has already been repaid:

$$MonthlyPayment = \frac{LoanAmount}{LoanLength} + \left(LoanAmount - CapitalRedemption\right) * \frac{APR}{100 * n} \quad (14)$$

Where *CapitalRedemption* (BDT) is the already repaid capital, 'n' is the number of months in the particular year when payment is expected. For example, if the loan length is 18 months, during the first year *n* equals 12, whereas in the second year *n* equals 6. In the current model application the loan length is set to 12 months (see Table S1.2). The loan characteristics are set based on the ESPA Deltas' household survey dataset.

The model also allows the households to postpone some of the payments if the money lender allows (see *LoanGracePeriod* in Table S1.2). The loan grace period parameter defines how many months the lender allows postponement of the payments. If the household is at 'food only' expenditure level (i.e. lowest material well-being level) and the loan grace period has not been used, the household can postpone the payment. If the loan grace period has been fully utilized already, the household has to sell some of its assets to pay for the loan. If the household has no assets left to sell, the lender has no other option but to postpone the payment again, until the household improves its financial situation.

S5.2 Land size calculation

The household land size is important as it defines the farm income in the calculations. The initial land size of each household archetype is determined from the ESPA Deltas' household survey dataset. In subsequently time steps, the land size is dynamically calculated based on (i) the initial land size, (ii) the total farm area within the union, (iii) the population size of the union, (iv) the mean household size and (v) the percent representation of each of household archetype within the union. The farm land changes are proportional to the changes of these input variables (e.g. if the population of a union doubles, the household land area halves and vice versa).

$$LandSize_{AR,t} = LandSize_{AR,t-1} * \frac{TotFarmArea_t}{TotFarmArea_{t-1}} * \frac{NoHhold_{AR,t-1}}{NoHhold_{AR,t}} \quad (15)$$

where *LandSize* (hectare) is the land owned by the specific household archetype (*AR*) in year *t*, *TotFarmArea* is the total agriculture area (hectare) in year *t*, *NoHhold* (number) is the number of households belonging to the specific archetype (AR) in year *t*.

Therefore, the model captures the generic characteristics of land consolidation, without explicitly modelling this process. In reality, Bangladesh has policies to cap the maximum land size owned by a person to 33 acres (~13 hectares). Land selling and purchasing is not modelled in the current version of the HEAP model, therefore, such an upper threshold (i.e. the 33 acres per person) is not considered. Land fragmentation due to inheritance however, is considered. In Bangladesh, land is considered the most important asset and safety-net and is thus households only sell it as the last resort. The user is allowed to constrain land fragmentation by defining a land protection threshold (see

LandProtectionTreshold in Table S1.2) that is considered to be the minimum farm size that can provide sustainable farm livelihoods. If this threshold is defined (e.g. 0.5 acres) and the simulated land size of a particular archetype falls below this threshold, the model forces the ‘surplus’ households (i.e. for whom no land would remain) to change their livelihoods from ‘farm owner’-based to an ‘off-farm’-based livelihood. The surplus households are uniformly allocated across the eligible, ‘off-farm-based’ archetypes. This ensures that land does not become too small and thus fragmented. This calculation, however, also means that the composition of archetypes in each union might change over time not only because of the Social-Ecological System (SES) changes, but also because of land fragmentation. Finally, the value of land is not considered in the total value of the assets, because it is assumed that for the above reasons (i.e. most important asset and safety net), land is preserved and kept at all expenditure [10].

S6. Initialization and calibration

The ESPA Deltas’ household survey interviewed 1586 households, of which 1478 was interviewed in all three seasons. These ‘complete’ household cases were used to define the archetypal households based on the levels of livelihood diversification across three seasons and the degree of land ownership. Then the average household characteristics (incomes, expenditures, savings) were calculated for each group for different time periods (1991, 1995, 2010, 2014) based on the HIES and ESPA Deltas’ household survey datasets (see Section 7 ‘Input data’). The household behaviours and model parameters were identified based on qualitative interviews conducted in the study area [4]. No stochasticity is considered in this version of the HEAP model (but this could be a future development).

Calibration was not done on the model parameters. This is because the model has numerous spatial simulation units (n=653 unions) and 36 household types in each union. There is no dataset that could be used to set HEAP up with such spatial detail and doing this manually (i.e. tampering with the inputs) could result in good results for the wrong reason. What was observed during the model tests is that the model settles automatically after some warm-up years even though the initial assets and cash savings are set differently. This is because the simulated households select their coping actions based on the imbalance of incomes, expenses and savings (cash and assets).

S7. Input data

This section explains all the input values, including the values of the user-defined variables mentioned above.

For this paper, all inputs are observations based: the 1991, 1995/6, 2010 HIES datasets [21] and the ESPA Deltas’ household survey dataset [3]. Thus, prices, wages are static inputs, based on observations for the historical period, and based on an assumed growth rate for the future period [22]. However, when the HEAP model, in subsequent publications, is applied as a component within the full ΔDIEM model, it uses estimates of environmental and (land and sea) productivity changes dynamically under an uncertain future [2, 23].

The HEAP model uses time series of income and direct livelihood expenditures (BDT/month) from (i) agriculture, (ii) aquaculture, (iii) fishing, (iv) forest good collection, (v) cottage industries, (vi) small service oriented businesses and salaried income jobs and (vii) remittances (Tables S1.4 – S1.6 in supporting data file: 'S3_Observed_inputs.xlsx'). The observed livelihood seasonality within the ESPA Deltas survey in 2014 was used to create historical seasonal livelihood income and expenditure data from the HIES datasets. In this paper, these parameters are derived from the above mentioned datasets to avoid introducing additional errors and uncertainties and thus to test the HEAP model validity with more confidence. The HEAP model also uses a time series of typical expenditure levels on food, essential house, non-essential, education, health, and social expenses (BDT/month) based on observations (Table S1.3 in supporting data file: 'S3_Observed_inputs.xlsx'). To achieve a continuous monthly input dataset, gaps in-between the time slices (1991, 1995, 2010, 2014) were estimated through interpolation. In addition to these time series, the user has to set a number of user-defined model parameters describing the socio-economic settings of the region and the general behaviour of the households (Table S1.2).

Table S1.1. Household archetypes based on the seasonally dominant livelihood and land size (based on the ESPA Deltas' household survey)

Archetype ID	Seasonally dominant livelihood (Season 1 – Season 2 – Season 3) (February-June; June-October; October-February)	Cumulative occurrence (%)	No. of households: Landless / Home-stead (LL:<0.5acres)	Small Land Owner (SLO: 0.5-2.5 acres)	Large Land Owner (LLO: >2.5 acres)	Sold/Grown Crop Ratio (LL – SLO – LLO)	Percent occurrence in each SES: Riverine / Charland	Sundarban dependent zone	Marine periphery	Irrigated agriculture	Rainfed agriculture	Saltwater shrimp aquaculture	Freshwater prawn aquaculture
1	SmallBusiness – SmallBusiness – SmallBusiness	19	103	157	24	53-42-45	29.92	21.53	27.13	35.34	36.20	25.68	28.15
2	CottageIndustry – CottageIndustry – CottageIndustry	33	132	72	0	53-43-na	18.11	22.92	19.38	13.79	23.93	25.00	20.00
3	FarmOwner – FarmOwner – FarmOwner	38	9	43	20	57-68-62	3.94	6.94	1.55	5.17	3.07	11.49	19.26
4	Fisher – Fisher – Fisher	42	42	20	0	49-50-na	24.41	4.17	9.30	0.86	3.07	2.03	1.48
5	CottageIndustry – FarmLabour – CottageIndustry	45	32	10	0	45-71-na	1.57	6.25	3.88	3.45	3.68	6.08	5.19
6	CottageIndustry – SmallBusiness – SmallBusiness	47	17	18	0	70-34-na	0.79	4.17	2.33	4.31	5.52	5.41	1.48
7	SmallBusiness – SmallBusiness – CottageIndustry	49	15	17	0	0-36-na	1.57	2.78	1.55	4.31	1.84	6.76	1.48
8	SmallBusiness – SmallBusiness – FarmOwner	52	0	25	7	na-70-52	2.36	2.08	6.98	0	3.68	2.70	2.22
9	FarmOwner – CottageIndustry – CottageIndustry	53	9	15	0	38-43-na	1.57	0.69	2.33	3.45	1.84	2.70	2.96
10	FarmOwner – noJob – FarmOwner	55	0	24	0	na-45-na	2.36	0.69	6.20	0.86	3.07	0.68	0.74
11	CottageIndustry – SmallBusiness – CottageIndustry	56	13	10	0	42-71-na	0.79	2.08	1.55	1.72	2.45	6.08	0
12	FarmOwner – SmallBusiness – FarmOwner	58	0	23	0	na-46-na	2.36	0.69	2.33	1.72	1.23	1.35	2.22
13	CottageIndustry – Fisher – CottageIndustry	59	14	8	0	52-55-na	2.36	4.86	4.65	0	0.61	0.68	1.48
14	FarmOwner – SmallBusiness – SmallBusiness	61	0	22	0	na-39-na	1.57	0	0	6.03	1.23	0.68	3.70
15	SmallBusiness – CottageIndustry – SmallBusiness	62	11	10	0	0-16-na	1.57	1.39	3.88	4.31	2.45	0.68	0.74
16	CottageIndustry – CottageIndustry – SmallBusiness	64	8	12	0	96-2-na	1.57	1.39	0.78	4.31	3.68	0.68	2.22
17	FarmLabour – CottageIndustry – CottageIndustry	65	20	0	0	12-na-na	0.79	0.69	1.55	4.31	1.23	0.68	3.70
18	FarmLabour – FarmLabour – CottageIndustry	66	20	0	0	0-na-na	0	2.78	0.78	4.31	0	0	2.22
19	SmallBusiness – CottageIndustry – CottageIndustry	68	8	11	0	0-28-na	2.36	2.78	3.10	1.72	1.23	0.68	0.74
20	Forest Good Collector	68	11	0	0	68-na-na	0	11.11	0.78	0	0	0	0

Table S1.2. Model parameter descriptions and values

Parameter	Description	Value
Initial cash	The household initial cash savings (BDT)	0
Initial assets	The value of the household initial assets (BDT)	0
Loan grace period	The number of months that a lender allows to postpone an instalment repayment	3
Target Expenditure Level	Expenditure level beyond no coping option is needed [1-6]	4
Official loan amount	The value of the official loan (BDT) that the household can access. (based on a 2014 review of loans in Bangladesh)	5000
Official loan length	The length of the official loan contract (months)	12
Official loan APR	The interest rate of the official loan (%)	28
Informal loan amount	The value of the informal loan (BDT) that the household can access. (based on a 2014 review of loans in Bangladesh)	8000
Informal loan length	The length of the informal loan contract (months)	12
Informal loan APR	The interest rate of the informal loan (%)	70
Max loan per season	The maximum number of loans that a household can take out in each season (based on the ESPA Deltas' household survey dataset)	1
Max loan per household	The maximum number of active loans that a household can have at any one time. (based on the ESPA Deltas' household survey dataset)	4
Waiting time UP	The minimum waiting time before the household decide to increase its expenditure level (months)	18
Max percent asset loss	The maximum asset loss per month that a household is ready to sacrifice to balance its finances and thus to maintain its well-being. (percent total) (based on the ESPA Deltas' household survey dataset)	30
Max BDT asset loss	The maximum asset loss per month that a household is ready to sacrifice to balance its finances and thus to maintain its well-being. (BDT) (based on the ESPA Deltas' household survey dataset)	20000
Min saved income	The percent of income that cannot be spent	0
Min remaining savings	The percent of cash savings that cannot be spent	30
Max zero expenditure	The maximum number of months per year when the household can get support from friend and family (i.e. zero expenditure)	3
Income drop	It is assumed that if a household cannot afford to pay the livelihood expenditures (needed to participate in the livelihood activity) in some months, they do not completely lose their income from that livelihood. This variable defines what percent of their potential income remains if no livelihood expenditures are paid.	40
Coping strategies	Coping strategies for each poverty class. Numbers demote to options (1-use cash savings; 2-sell assets; 3-get official loan; 4-get informal loan; 5-drop expenditure). The order shows priority.<br>Expenditure level 1 (poorest): 1,5,4,2 (no access to official loan)<br>Expenditure levels 2-3 (poorer): 1, 5, 2, 4, 3<br>Expenditure levels 4-5 (less poor): 1, 3, 4, 2	
Land protection threshold	The land size below which the land would become too small to support sufficient FarmOwner livelihood (acres).	0.5

Table S1.3. Expected economic changes in coastal Bangladesh between 2015 and 2030 (percent change) under a Business As Usual scenario [22, 23].

**Economic input variable - DDIEM**	**Percent change by 2030 under the BAU scenario**
Cost of agriculture inputs (fertiliser BDT/kg, pesticide BDT/ha, seed BDT/kg, aquaculture feed BDT/ha, post larvae or fishling BDT/individual)	10
Cost to run a cottage industry or small service business (BDT/month)	0
Cost to keep livestock/poultry (BDT/month)	10
Cost of fishing, Forest collection (BDT/month)	10
Cost of diesel (BDT/gallon)	10
Market (selling) price of agriculture crops, fish and aquaculture crops (e.g. shrimp) (BDT/kg)	10
Income from forest goods (honey, fruits, timber, etc.) (BDT/month)	-10
Income from Manufacturing, Services and Livestock/Poultry sectors (BDT/month)	110
Daily wage (without food) (BDT/day)	10
Remittances (BDT/month)	30
Household expenses (BDT/month)	10
Land rent cost (farming) (BDT/month)	10
Purchase Power Parity (PPP) exchange rate for Bangladesh (-)	0
USD/BDT exchange rate (-)	0

S8. References

1. Grimm V, Berger U, DeAngelis DL, Polhill JG, Giske J, Railsback SF. The ODD protocol: A review and first update. Ecological Modelling. 2010;221(23):2760-8. doi:http://dx.doi.org/10.1016/j.ecolmodel.2010.08.019
2. Nicholls RJ, Hutton CW, Lázár AN, Allan A, Adger WN, Adams H, et al. Integrated assessment of social and environmental sustainability dynamics in the Ganges-Brahmaputra-Meghna delta, Bangladesh. Estuarine and coastal shelf science. 2016;183:370–81
3. Adams H, Adger WN, Ahmad S, Ahmed A, Begum D, Lázár AN, et al. Spatial and temporal dynamics of multidimensional well-being, livelihoods and ecosystem services in coastal Bangladesh. Scientific Data. 2016;3:160094. doi:10.1038/sdata.2016.94
4. Adams H, Adger WN, Ahmad S, Ahmed A, Begum D, Matthews Z, et al. Spatial and temporal dynamics of multidimensional well-being, livelihoods and ecosystem services in coastal Bangladesh. Colchester, Essex: UK Data Archive. 10.5255/UKDA-SN-8521792016
5. Narayan D, Chambers R, Shah M, Petesch P. Global synthesis: Consultations with the Poor. Washington, D.C.: World Bank; 1999.
6. Falkingham J, Namazie C. Measuring health and poverty: a review of approaches to identifying the poor. DFID Health Systems Resource Centre; 2002.
7. Stark O, Bloom DE. The New Economics of Labor Migration. The American Economic Review. 1985;75(2):173-8
8. Hulme D, Shepherd A. Conceptualizing Chronic Poverty. World Development. 2003;31(3):403-23. doi:10.1016/S0305-750X(02)00222-X
9. Rahman PMM, Matsui N, Ikemoto Y. Dynamics of Poverty in Rural Bangladesh. Tokyo: Springer; 2013. doi:10.1007/978-4-431-54285-8
10. Toufique KA, Turton C. Hands not land: How livelihoods are changing in rural Bangladesh. Bangladesh Institute of Development Studies, ISBN: 1-86192-492-52002. 136 p
11. Pickett KE, Wilkinson RG. Income inequality and health: a causal review. Social Science & Medicine. 2015;128:316-26
12. Adger WN. Vulnerability. Global Environmental Change. 2006;16(3):268-81
13. Blaikie P, Cannon T, Davis I, Wisner B. At Risk: Natural Hazards, People's Vulnerability and Disasters (2nd edition). London: Routledge2004.
14. Lenhardt A, Shepherd A. What has happened to the poorest 50%? University of Manchester, Manchester: Brooks World Poverty Institute; 2013.
15. Sabates R, Hossain A, Lewin KM. School drop out in Bangladesh: Insights using panel data. International Journal of Educational Development. 2013;33(3):225-32
16. Hamid SA, Ahsan SM, Begum A. Disease-specific impoverishment impact of out-of-pocket payments for health care: evidence from rural Bangladesh. Applied health economics and health policy. 2014;12(4):421-33
17. Massey DS, Arango J, Hugo G, Kouaouci A, Pellegrino A, Taylor JE. Theories of international migration: A review and appraisal. Population and development review. 1993;19(3):431-66

18. Alkire S, Conconi A, Seth S. Measuring Destitution in Developing Countries: An Ordinal Approach for Identifying Linked Subsets of the Multidimensionally Poor. OPHI Research in Progress; 2014.
19. Sultana N, Rayhan MI. Coping strategies with floods in Bangladesh: an empirical study. Nat Hazards. 2012;64(2):1209-18. doi:10.1007/s11069-012-0291-5
20. Davis P. The Trappings of Poverty: The Role of Assets and Liabilities in Socio-Economic Mobility in Rural Bangladesh. SSRN: Chronic Poverty Research Centre 2011.
21. BBS. Report of the Household Income & Expenditure Survey 1991. Bangladesh Bureau of Statistics, Statistical Division, Ministry of Planning; 1991.
22. Hunt A. Future scenarios of economic development. In: Nicholls JR, Hutton CW, Adger WN, Hanson S, Rahaman M, Salehin M, editors. Ecosystem Services For Well-Being In Deltas: Integrated Assessment For Policy Analysis: Palgrave, ISBN 978-3-319-71092-1; 2018
23. Lázár AN, Payo A, Adams H, Ahmed A, Allan A, Akanda AR, et al. Integrative analysis applying the Delta Dynamic Integrated Emulator Model in south-west coastal Bangladesh. In: Nicholls RJ, Hutton CW, Adger WN, Hanson S, Rahaman M, Salehin M, editors. Ecosystem Services For Well-Being In Deltas: Integrated Assessment For Policy Analysis: Palgrave, ISBN 978-3-319-71092-1; 2018
