# Supplementary material for: Modelling household well-being and poverty trajectories: An application to coastal Bangladesh
Source: PLoS One. 2020 Sep 4;15(9):e0238621. doi: 10.1371/journal.pone.0238621 (PMC7473571; doi:10.1371/journal.pone.0238621)

Supporting Information (2) for

Modelling household well-being and poverty trajectories: an application to coastal Bangladesh

Attila N. Lázár[1]*, Helen Adams[2], W. Neil Adger[3], Robert J. Nicholls[4]

[1] Department of Geography and Environmental Science, University of Southampton, Southampton, United Kingdom

[2] Department of Geography, King's College London, Strand Campus, London, United Kingdom

[3] Department of Geography, College of Life and Environmental Sciences, University of Exeter, Exeter, United Kingdom

[4] Tyndall Centre for Climate Change Research, University of East Anglia, Norwich, United Kingdom

*** Corresponding author:**

Email: a.lazar@soton.ac.uk (ANL)

Contents of this file

Figure S2.1: Simulated intra and inter-seasonal livelihood diversification and income-levels (thousand BDT/month) of the household livelihood archetypes. 2-4
Figure S2.2: Simulated expenditure-based well-being level of the household livelihood archetypes. 5-7
Figure S2.3: Poverty trajectories of household archetypes. 8-12
Figure S2.4. Scatter plots of household characteristics. 13

Supporting Information (Files uploaded separately)

S1 file: HEAP (S1_HEAP.pdf)
S2 file: All plots (S2_All_plots.pdf)
S3 file: Observed inputs (S3_Observed_inputs.xlsx)
S4 file: Figure supporting data (S4_Figure_Supporting_Data.xlsx)

Figure S2.1. Simulated intra and inter-seasonal livelihood diversification and income-levels (thousand BDT/month) of the household livelihood archetypes (only shown for the last 2 years of simulation to be able to see the details). AR: archetype number; LL: Landless; SLO: Small Land Owner; LLO: Large Land Owner.

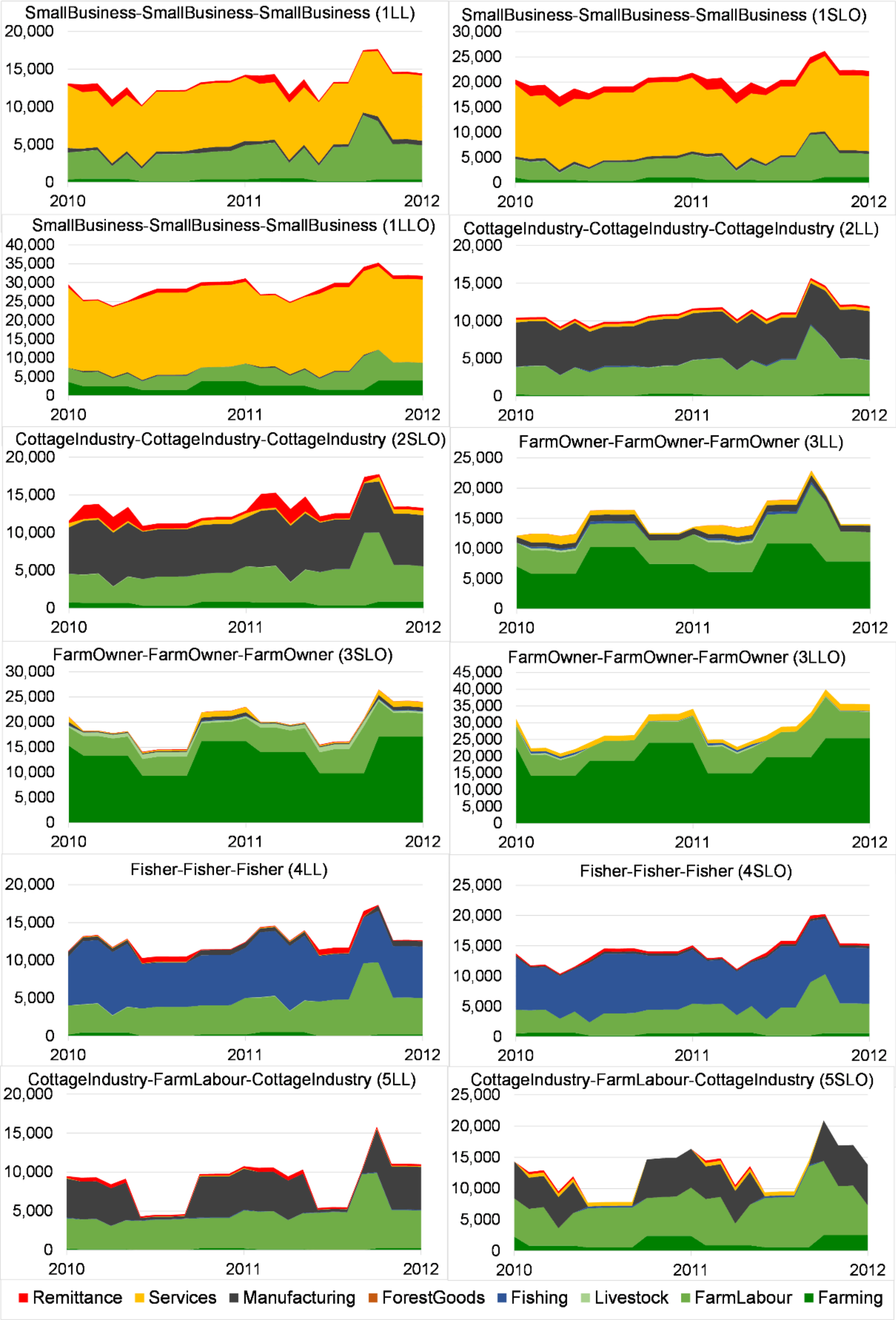

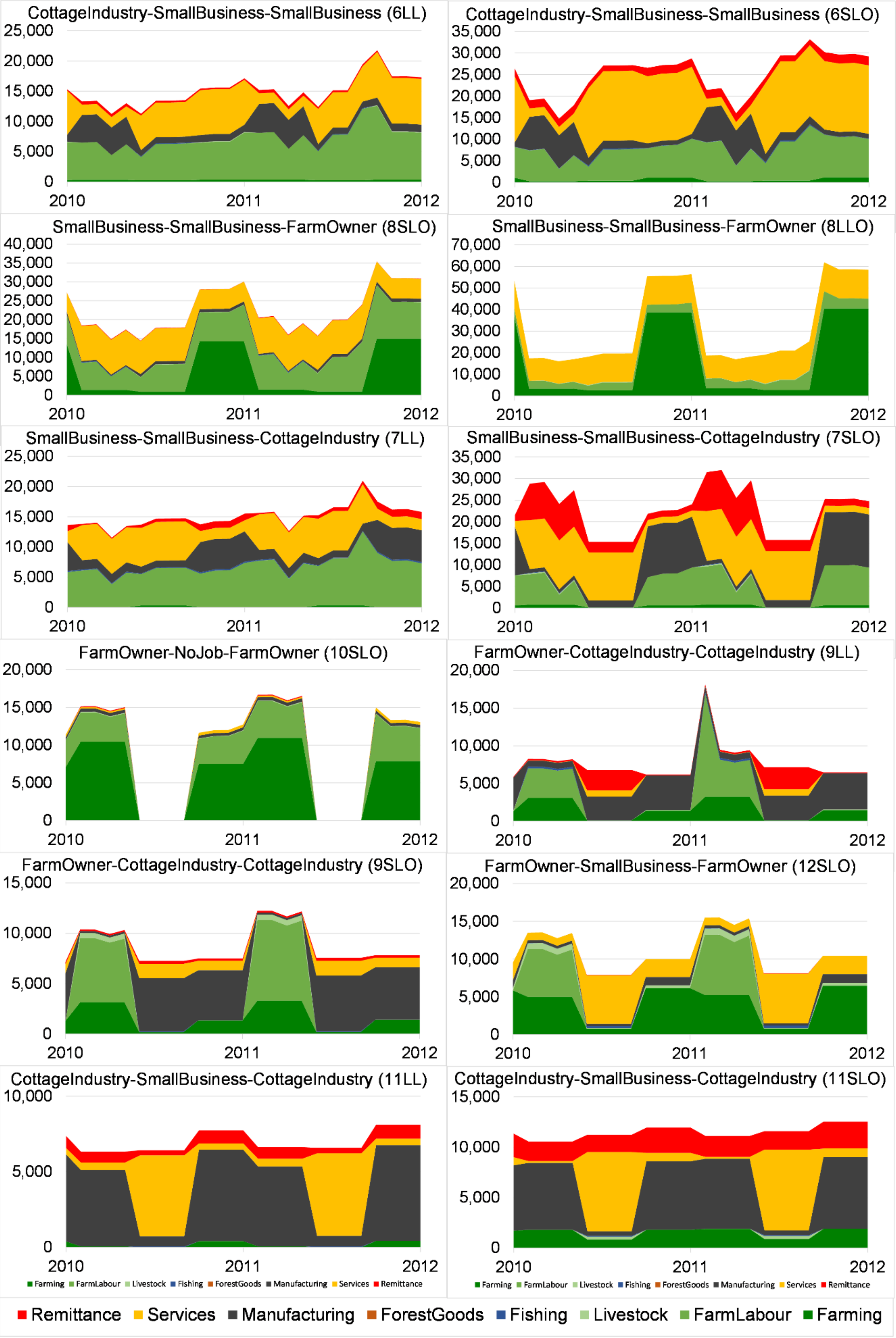
CottageIndustry-SmallBusiness-SmallBusiness (6LL)
CottageIndustry-SmallBusiness-SmallBusiness (6SLO)
SmallBusiness-SmallBusiness-FarmOwner (8SLO)
SmallBusiness-SmallBusiness-FarmOwner (8LLO)
SmallBusiness-SmallBusiness-CottageIndustry (7LL)
SmallBusiness-SmallBusiness-CottageIndustry (7SLO)
FarmOwner-NoJob-FarmOwner (10SLO)
FarmOwner-CottageIndustry-CottageIndustry (9LL)
FarmOwner-CottageIndustry-CottageIndustry (9SLO)
FarmOwner-SmallBusiness-FarmOwner (12SLO)
CottageIndustry-SmallBusiness-CottageIndustry (11LL)
CottageIndustry-SmallBusiness-CottageIndustry (11SLO)
2010
2011
2012
Farming FarmLabour Livestock Fishing ForestGoods Manufacturing Services Remittance
Remittance Services Manufacturing ForestGoods Fishing Livestock FarmLabour Farming

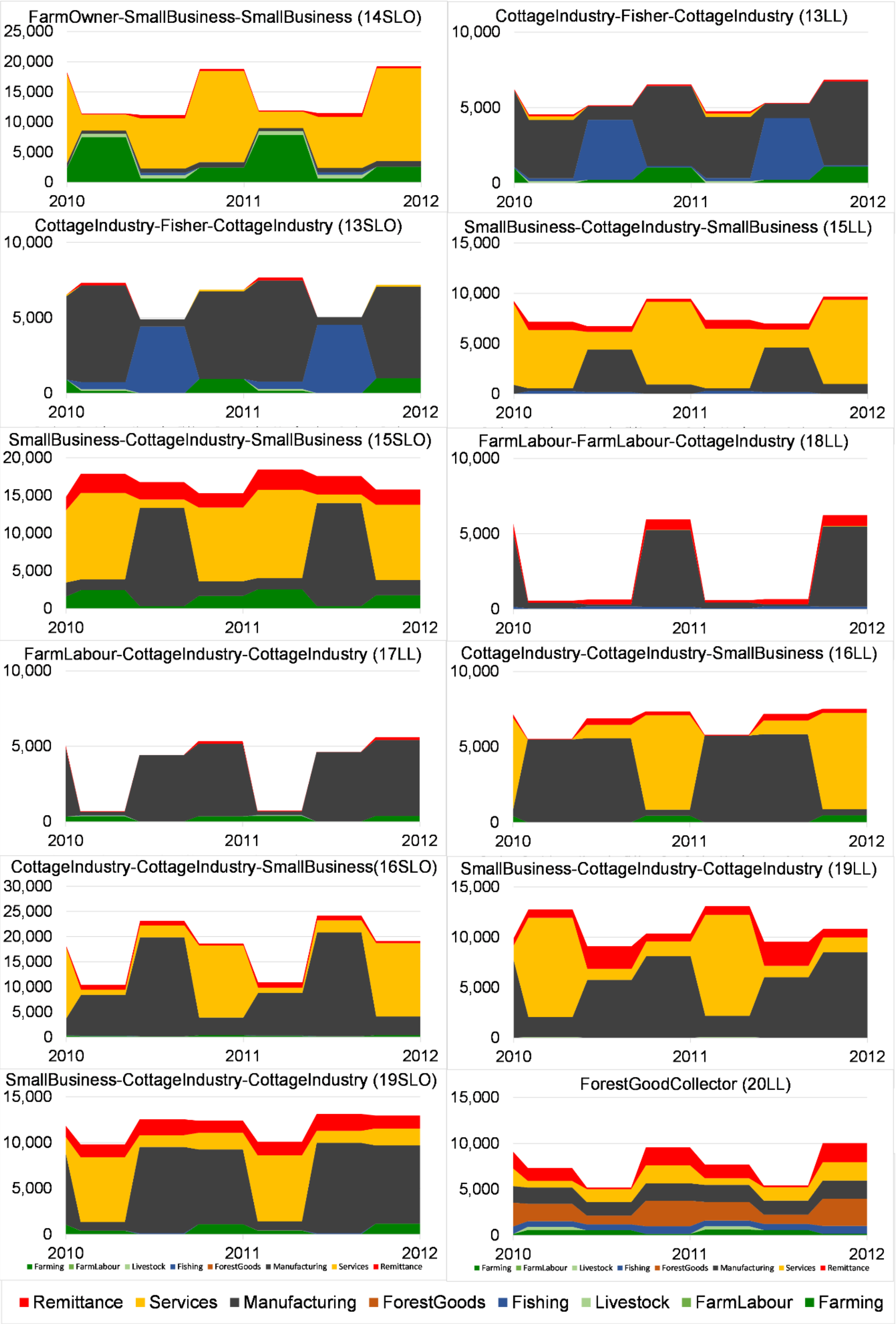
FarmOwner-SmallBusiness-SmallBusiness (14SLO)
CottageIndustry-Fisher-CottageIndustry (13LL)
CottageIndustry-Fisher-CottageIndustry (13SLO)
SmallBusiness-CottageIndustry-SmallBusiness (15LL)
SmallBusiness-CottageIndustry-SmallBusiness (15SLO)
FarmLabour-FarmLabour-CottageIndustry (18LL)
FarmLabour-CottageIndustry-CottageIndustry (17LL)
CottageIndustry-CottageIndustry-SmallBusiness (16LL)
CottageIndustry-CottageIndustry-SmallBusiness(16SLO)
SmallBusiness-CottageIndustry-CottageIndustry (19LL)
SmallBusiness-CottageIndustry-CottageIndustry (19SLO)
ForestGoodCollector (20LL)
2010
2011
2012
Remittance
Services
Manufacturing
ForestGoods
Fishing
Livestock
FarmLabour
Farming

Figure S2.2. Simulated expenditure-based well-being level of the household livelihood archetypes. Solid black line represents the study area mean value, whereas the grey area highlights the ±1 standard deviation from the mean. AR: archetype number; LL: Landless; SLO: Small Land Owner; LLO: Large Land Owner.

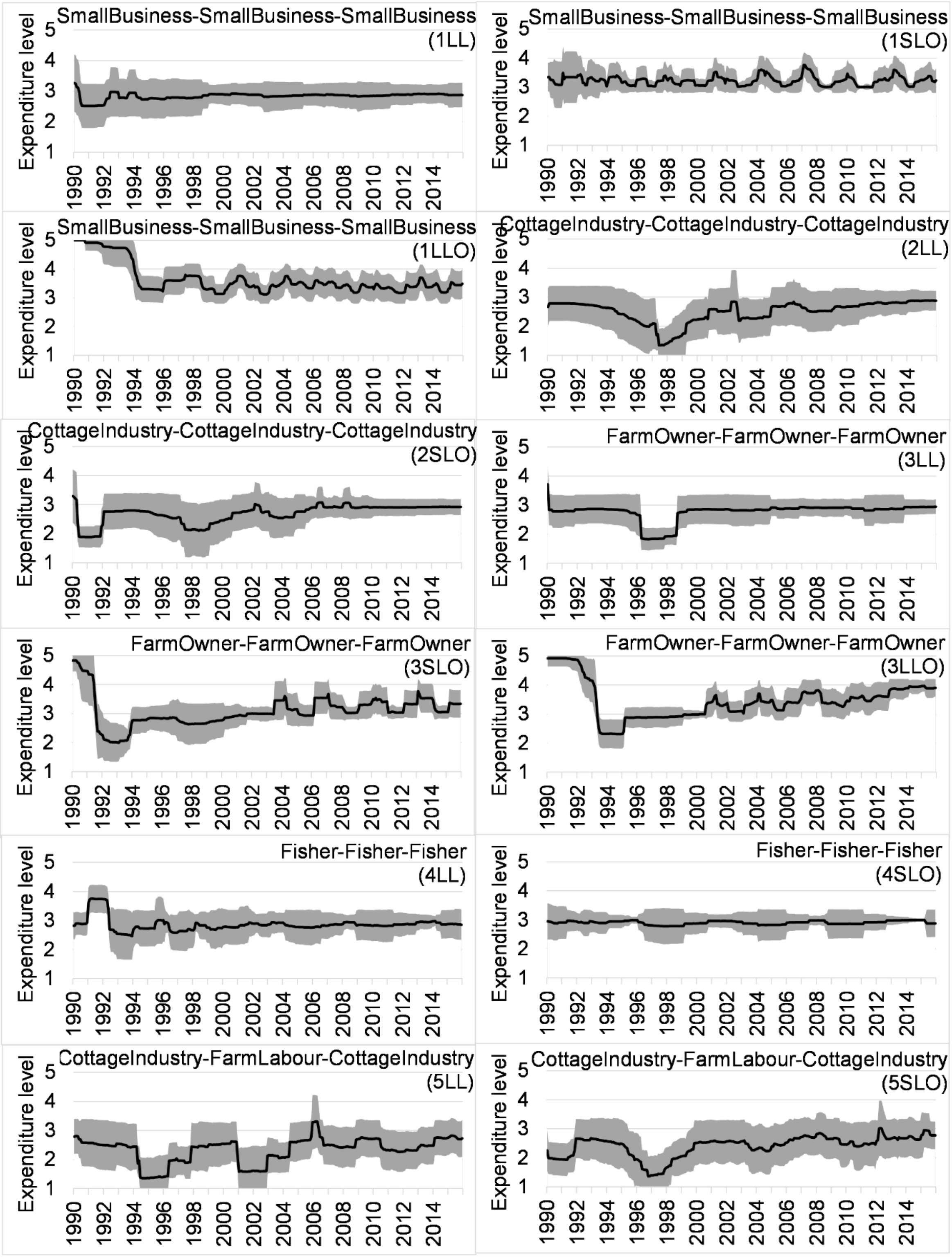

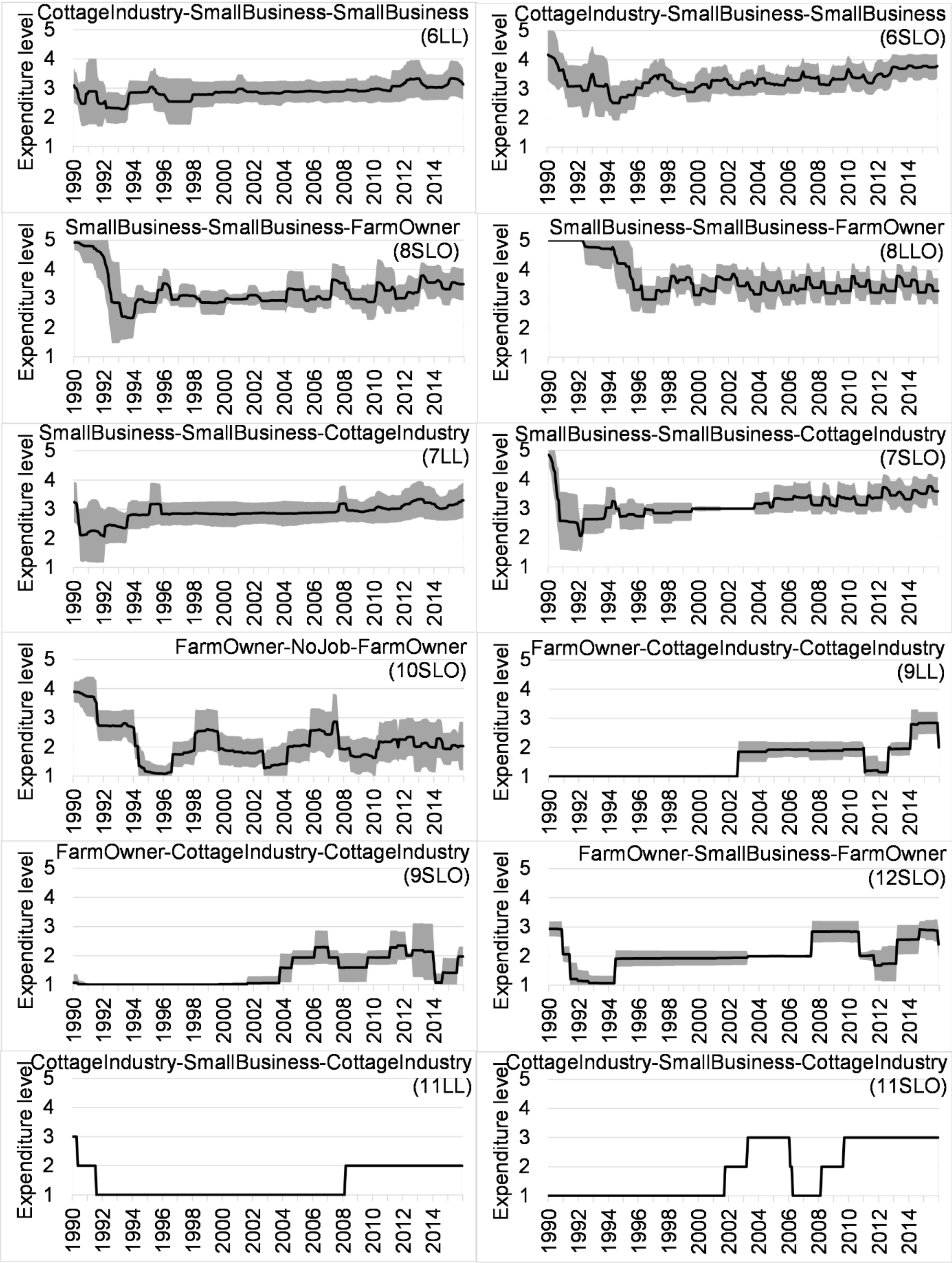

CottageIndustry-SmallBusiness-SmallBusiness (6LL)
CottageIndustry-SmallBusiness-SmallBusiness (6SLO)
SmallBusiness-SmallBusiness-FarmOwner (8SLO)
SmallBusiness-SmallBusiness-FarmOwner (8LLO)
SmallBusiness-SmallBusiness-CottageIndustry (7LL)
SmallBusiness-SmallBusiness-CottageIndustry (7SLO)
FarmOwner-NoJob-FarmOwner (10SLO)
FarmOwner-CottageIndustry-CottageIndustry (9LL)
FarmOwner-CottageIndustry-CottageIndustry (9SLO)
FarmOwner-SmallBusiness-FarmOwner (12SLO)
CottageIndustry-SmallBusiness-CottageIndustry (11LL)
CottageIndustry-SmallBusiness-CottageIndustry (11SLO)
Expenditure level
1
2
3
4
5
1990
1992
1994
1996
1998
2000
2002
2004
2006
2008
2010
2012
2014

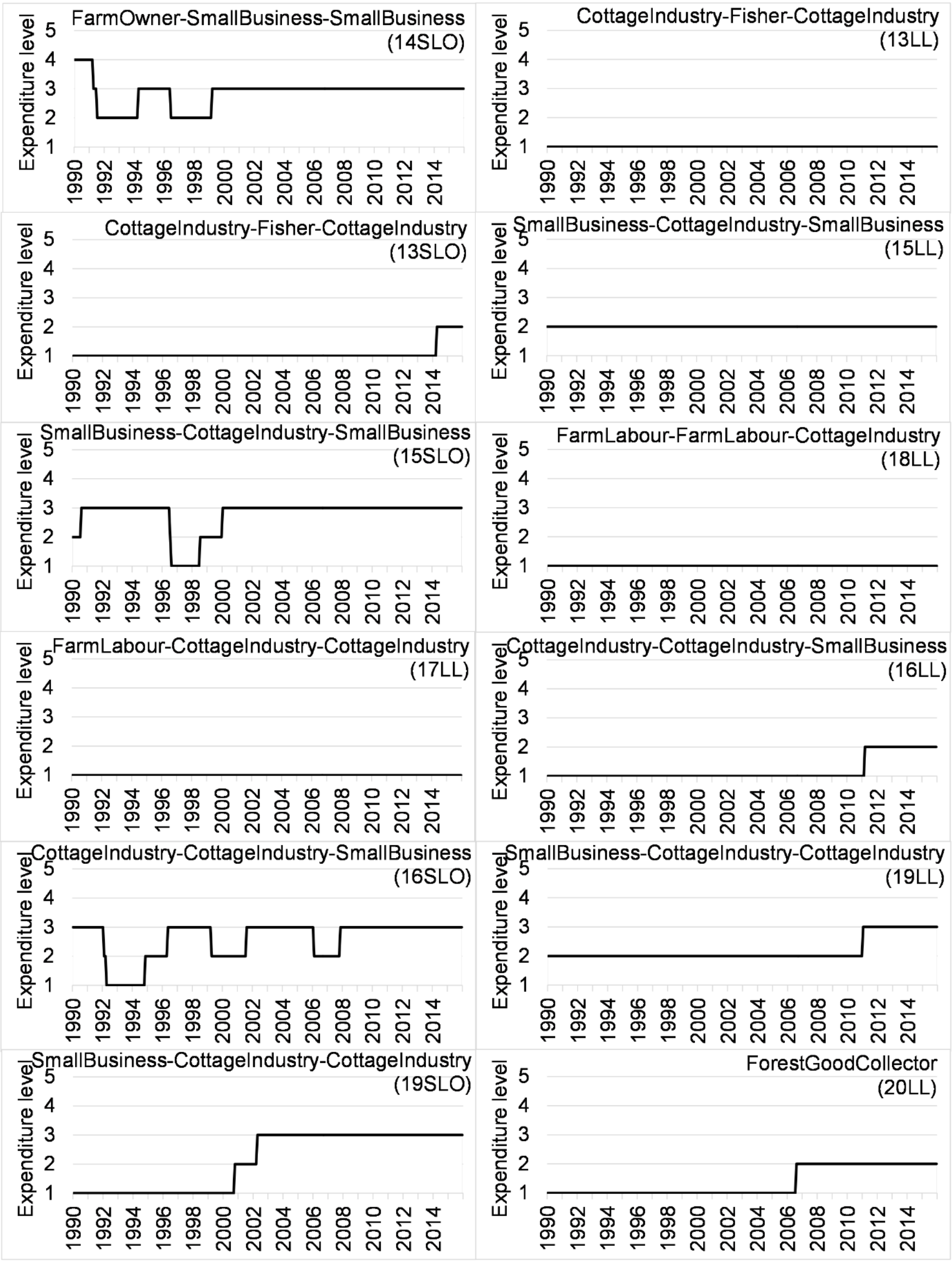
FarmOwner-SmallBusiness-SmallBusiness (14SLO)
CottageIndustry-Fisher-CottageIndustry (13LL)
CottageIndustry-Fisher-CottageIndustry (13SLO)
SmallBusiness-CottageIndustry-SmallBusiness (15LL)
SmallBusiness-CottageIndustry-SmallBusiness (15SLO)
FarmLabour-FarmLabour-CottageIndustry (18LL)
FarmLabour-CottageIndustry-CottageIndustry (17LL)
CottageIndustry-CottageIndustry-SmallBusiness (16LL)
CottageIndustry-CottageIndustry-SmallBusiness (16SLO)
SmallBusiness-CottageIndustry-CottageIndustry (19LL)
SmallBusiness-CottageIndustry-CottageIndustry (19SLO)
ForestGoodCollector (20LL)
Expenditure level
1
2
3
4
5
1990
1992
1994
1996
1998
2000
2002
2004
2006
2008
2010
2012
2014

Figure S2.3. Poverty trajectories of household archetypes. The shade of the Calorie intake, Education and Assets index indicate the severity of the deprivation (the darker, the more deprived). The red dashed line marks the multidimensional poverty line. (AR: archetype number; LL: Landless; SLO: Small Land Owner; LLO: Large Land Owner; MPI: Multidimensional Poverty Index).

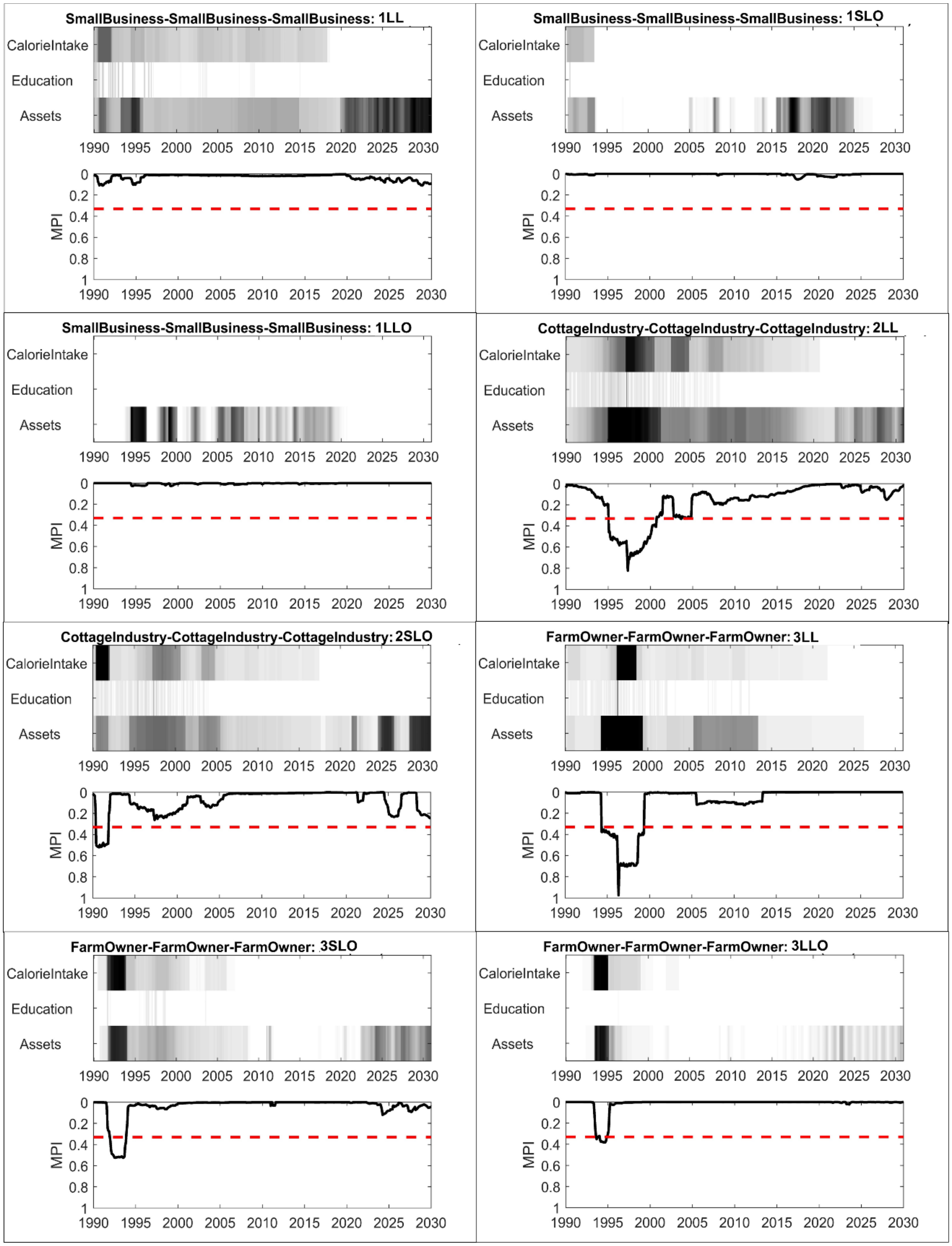

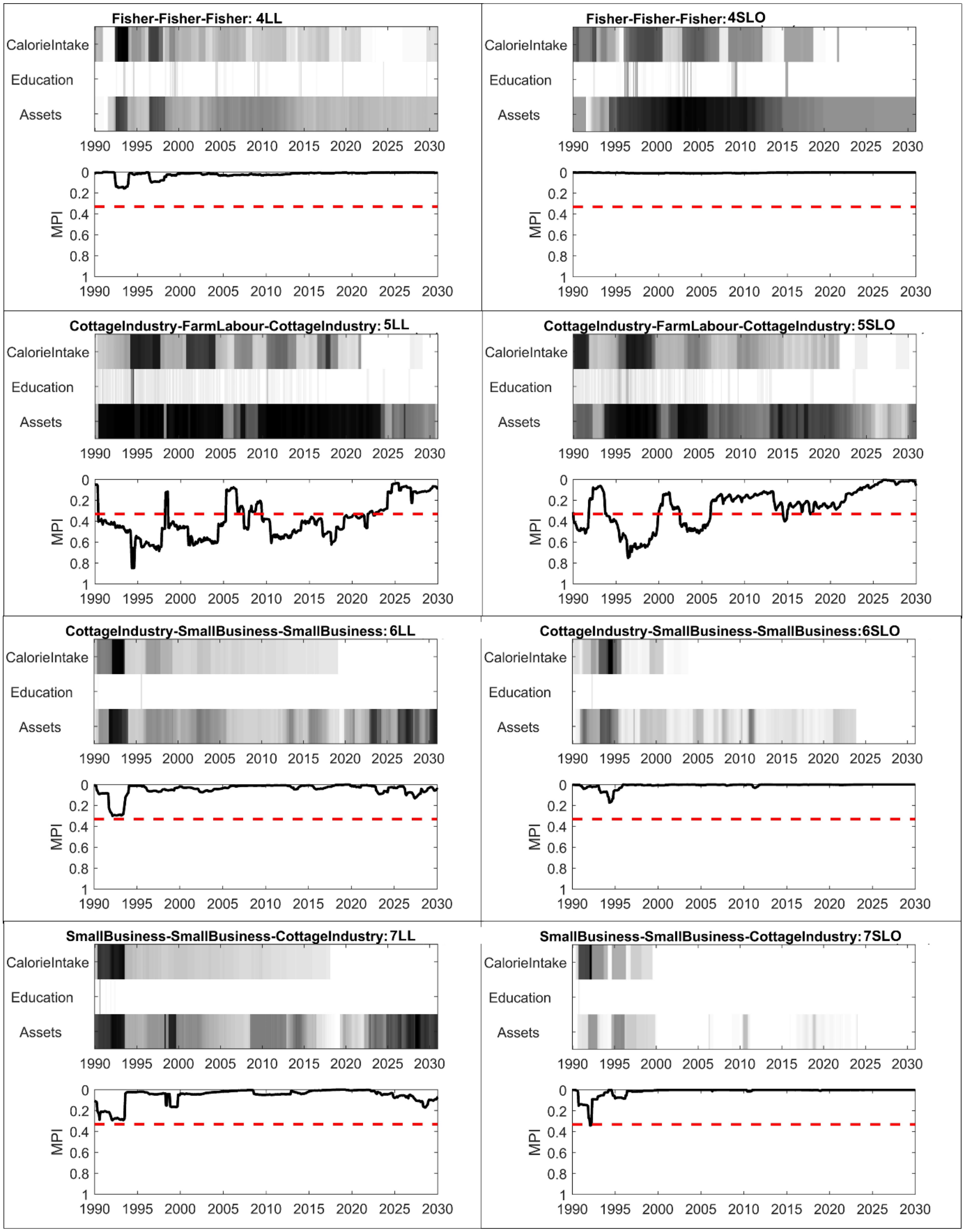

Fisher-Fisher-Fisher: 4LL
Fisher-Fisher-Fisher: 4SLO
CottageIndustry-FarmLabour-CottageIndustry: 5LL
CottageIndustry-FarmLabour-CottageIndustry: 5SLO
CottageIndustry-SmallBusiness-SmallBusiness: 6LL
CottageIndustry-SmallBusiness-SmallBusiness: 6SLO
SmallBusiness-SmallBusiness-CottageIndustry: 7LL
SmallBusiness-SmallBusiness-CottageIndustry: 7SLO
CalorieIntake
Education
Assets
MPI

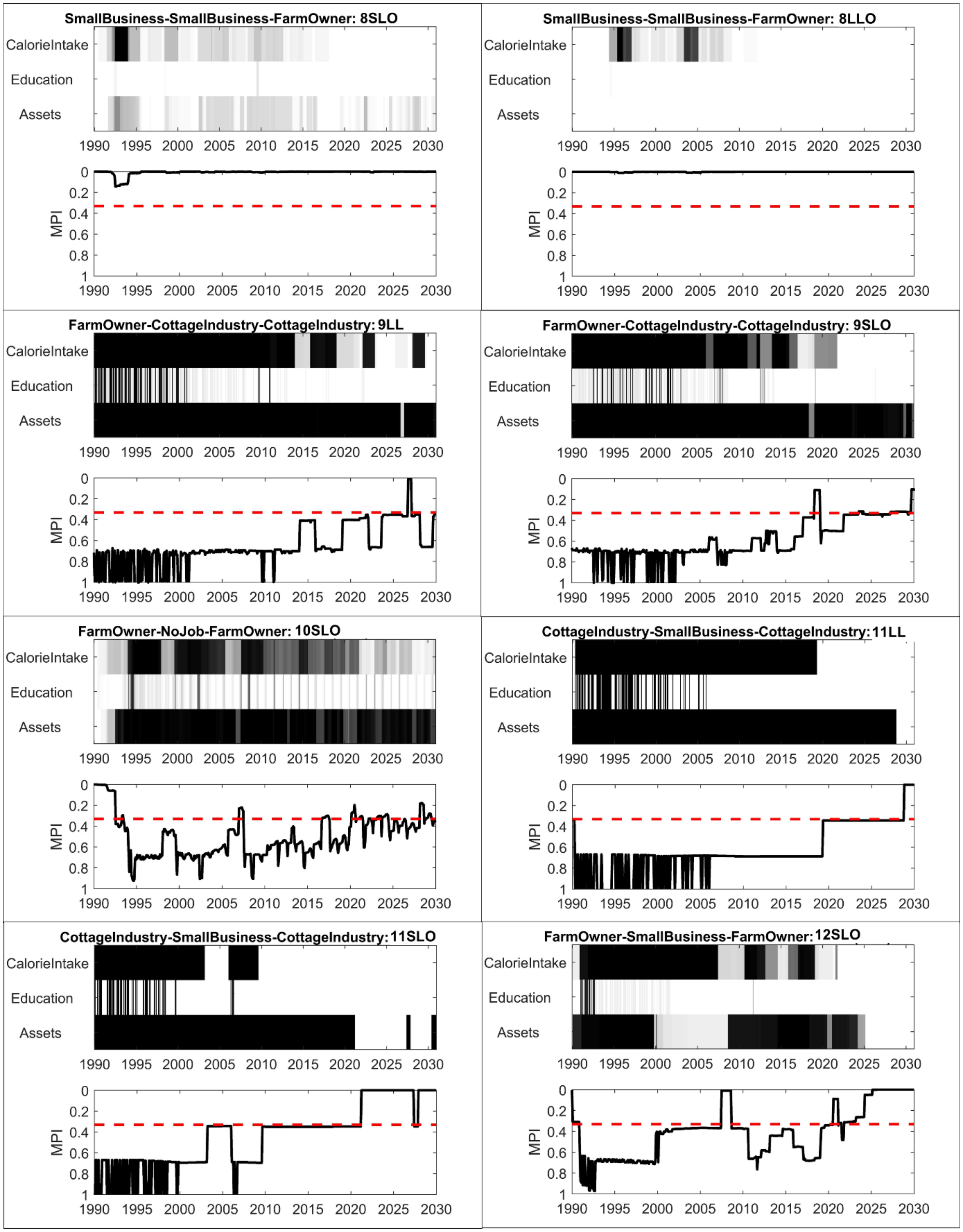

SmallBusiness-SmallBusiness-FarmOwner: 8SLO
SmallBusiness-SmallBusiness-FarmOwner: 8LLO
FarmOwner-CottageIndustry-CottageIndustry: 9LL
FarmOwner-CottageIndustry-CottageIndustry: 9SLO
FarmOwner-NoJob-FarmOwner: 10SLO
CottageIndustry-SmallBusiness-CottageIndustry: 11LL
CottageIndustry-SmallBusiness-CottageIndustry: 11SLO
FarmOwner-SmallBusiness-FarmOwner: 12SLO
CalorieIntake
Education
Assets
MPI
0
0.2
0.4
0.6
0.8
1
1990
1995
2000
2005
2010
2015
2020
2025
2030

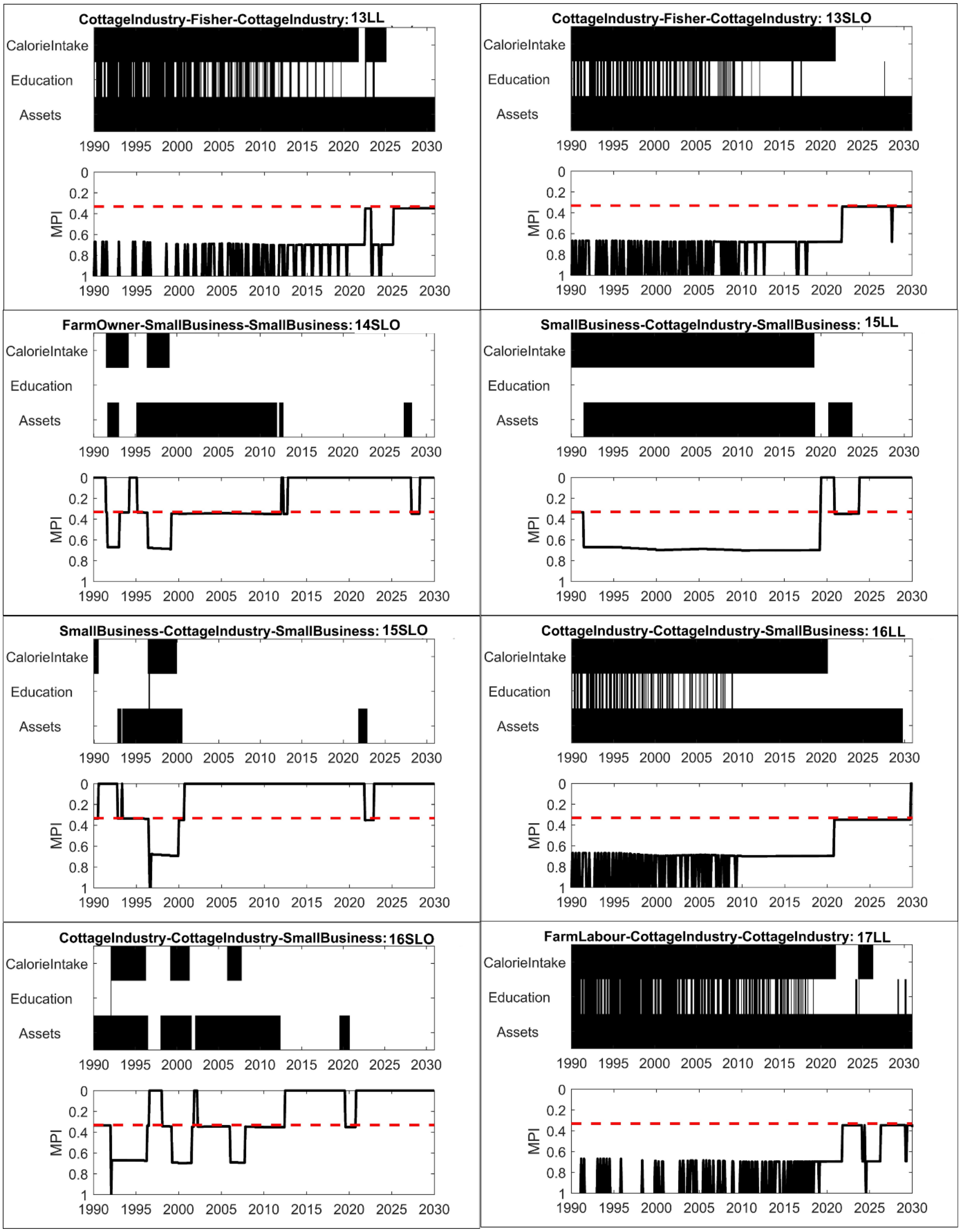
CottageIndustry-Fisher-CottageIndustry: 13LL
CottageIndustry-Fisher-CottageIndustry: 13SLO
FarmOwner-SmallBusiness-SmallBusiness: 14SLO
SmallBusiness-CottageIndustry-SmallBusiness: 15LL
SmallBusiness-CottageIndustry-SmallBusiness: 15SLO
CottageIndustry-CottageIndustry-SmallBusiness: 16LL
CottageIndustry-CottageIndustry-SmallBusiness: 16SLO
FarmLabour-CottageIndustry-CottageIndustry: 17LL
CalorieIntake
Education
Assets
MPI
1990 1995 2000 2005 2010 2015 2020 2025 2030
0 0.2 0.4 0.6 0.8 1

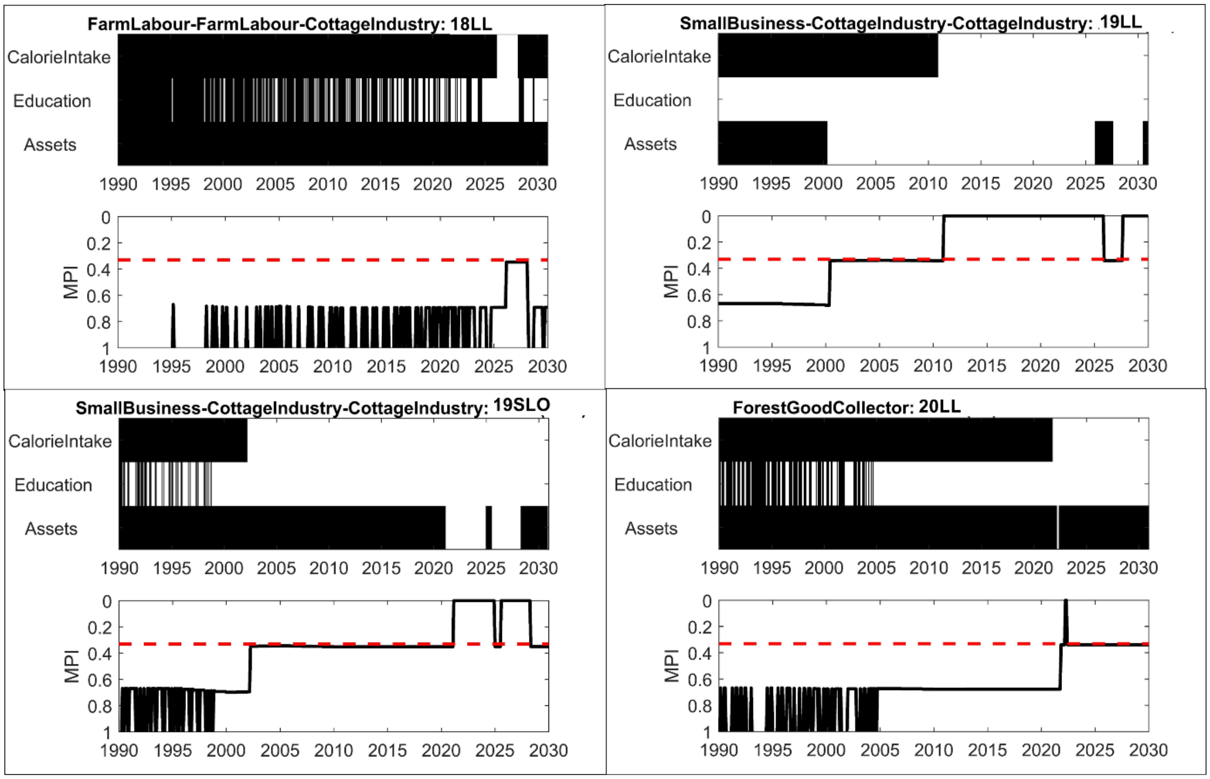
FarmLabour-FarmLabour-CottageIndustry: 18LL
CalorieIntake
Education
Assets
MPI
SmallBusiness-CottageIndustry-CottageIndustry: 19LL
SmallBusiness-CottageIndustry-CottageIndustry: 19SLO
ForestGoodCollector: 20LL

Figure S2.4. Scatter plots of some household characteristics. Plotted characteristics represent the mean simulated value. Dots are household archetypes. Variable dot size represents the number of households. Uniform dot size represents individual household archetypes. (LL: Landless; SLO: Small Land Owner; LLO: Large Land Owner; MPI: Multidimensional Poverty Index)

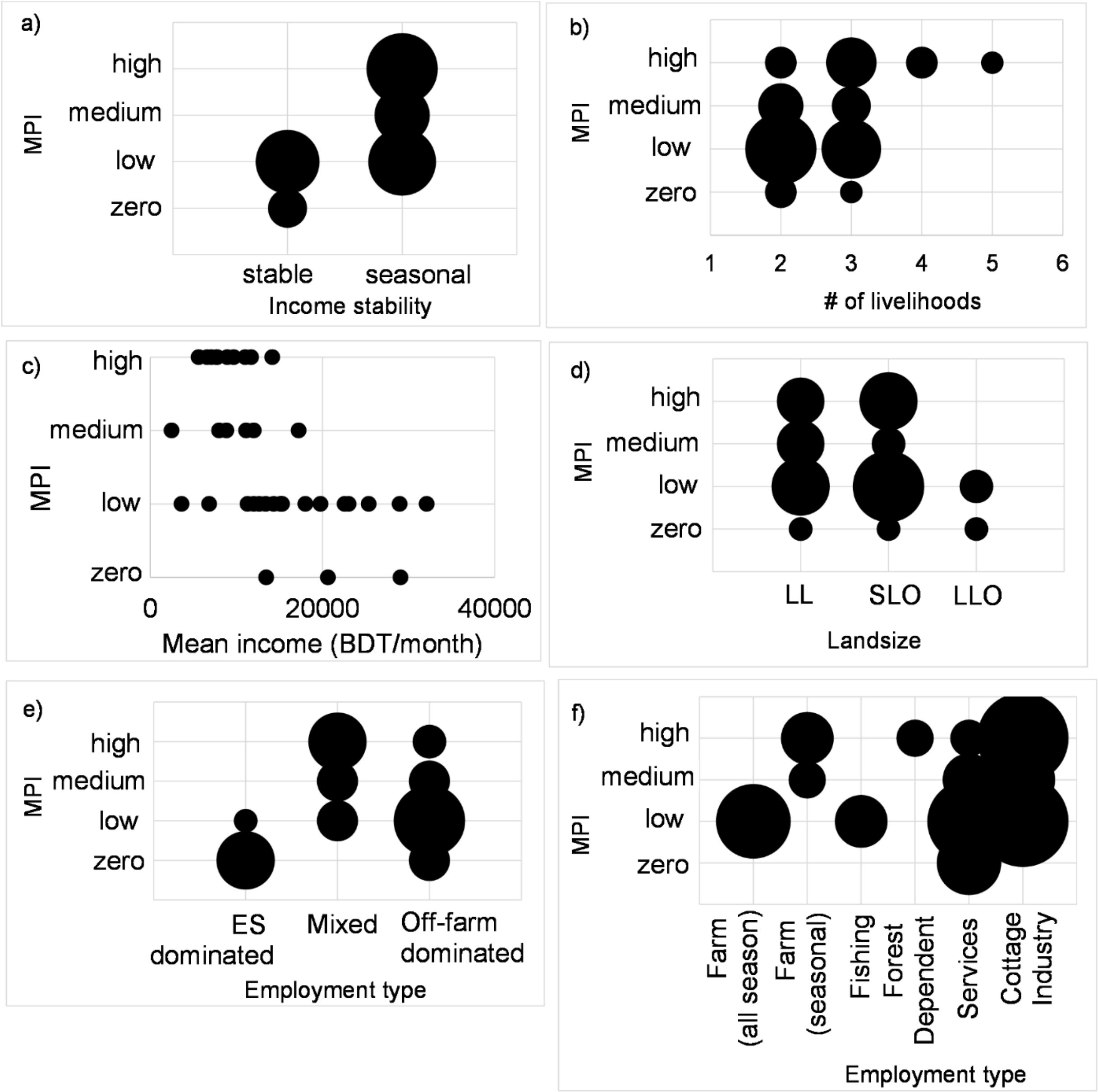

Supplement: S2 File — (PDF) [file pone.0238621.s002.pdf]
